# Smart Carriers and Nanohealers: A Nanomedical Insight on Natural Polymers

**DOI:** 10.3390/ma10080929

**Published:** 2017-08-10

**Authors:** Sreejith Raveendran, Ankit K. Rochani, Toru Maekawa, D. Sakthi Kumar

**Affiliations:** Bio Nano Electronics Research Centre, Graduate School of Interdisciplinary New Science, Toyo University, Saitama 350-8585, Japan; sreejithr84@gmail.com (S.R.); anrochani@gmail.com (A.K.R.); maekawa@toyo.jp (T.M.)

**Keywords:** biopolymers, nanoscience, polymers, drug delivery, tissue engineering, nanoparticles

## Abstract

Biodegradable polymers are popularly being used in an increasing number of fields in the past few decades. The popularity and favorability of these materials are due to their remarkable properties, enabling a wide range of applications and market requirements to be met. Polymer biodegradable systems are a promising arena of research for targeted and site-specific controlled drug delivery, for developing artificial limbs, 3D porous scaffolds for cellular regeneration or tissue engineering and biosensing applications. Several natural polymers have been identified, blended, functionalized and applied for designing nanoscaffolds and drug carriers as a prerequisite for enumerable bionano technological applications. Apart from these, natural polymers have been well studied and are widely used in material science and industrial fields. The present review explains the prominent features of commonly used natural polymers (polysaccharides and proteins) in various nanomedical applications and reveals the current status of the polymer research in bionanotechnology and science sectors.

## 1. Introduction

Bionanoscience refers to a postmodern field of study that groups biotechnology and nanotechnology into a single platform, focusing on the controlled manipulation and study of devices, structures, molecules and mechanisms on the nanoscale. Researchers have found it increasingly significant, as there are innumerable issues in science and technology that macromolecular concepts cannot solve. Nanoscale materials are gaining relevance in several biomedical applications, including tissue engineering, cancer therapy, drug delivery, hyperthermia, photoluminescence, bioimaging, etc. due to their structure, size, and surface area. Degradable polymer-based nanomaterials are highly preferred for a wide range of applications, including nanomedicine, water purification, microelectronics, agriculture and environmental protection. The biocompatible polymeric nanoparticles help to overcome problems related to stability, cytotoxicity, biocompatibility, biodegradability, etc. They improve the efficacy of drugs and reduce side effects via direct targeting and site-specific sustained/controlled delivery with enhanced cellular uptake [1,2]. Inorganic nanoparticles synthesized for biomedical applications can be embedded in biocompatible polymers to reduce the toxicity and enhance cellular acceptance. The flexibility of designing polymer systems that can respond to external stimuli such as pH, ultrasound and magnetic field has given rise to smart therapy. Such shape-changing smart polymers were used in surgeries for insertion of self-inflating bulky medical devices.

Polymers have been under study for decades, and many critical reviews have been published based on the glowing accounts of polymers and their uses. They are widely used for several nanomedical and bioscience applications, including tissue engineering, regeneration, and drug delivery, based on their physicochemical, biological and pharmacological properties. Cellular regeneration/reconstruction or engineering plays a critical role in the healing process from pathological conditions. Factors that affect the cellular regeneration or wound healing and healthy tissue reconstruction have been extensively reviewed by Guo, S et al. [3]. Some of the major factors that affect the wound healing process and tissue regeneration include inflammatory response, infection, deregulation of proteases, impairments in the localization of progenitor cells at the site of injury and inadequate localized angiogenesis [4]. The cellular mechanism of wound healing is an extremely complex process that simultaneously orchestrates a number of cellular subtypes (Table 1). Chronic impairment in wound healing or tissue regeneration can occasionally lead to loss of cellular senescence and/or development of cancers. Recent studies have suggested that improper wound healing and tissue regeneration can also lead to several malignancies [5]. Moreover, chronic inflammation caused due to improper tissue regeneration can give rise to stressful microenvironments facilitating the formation of cancer stem cells (CSCs). This shows that improper cellular regeneration/wound healing is an important biomedical problem to be addressed. Biomaterials that are used for development of wound dressings play an essential role in tissue engineering, regeneration and wound healing. Novel and smart-biomaterials are required to enhance the basic properties of wound dressings that include anti-infective, antioxidative, adhesive, migrating and proliferative nature of growing cells.

**Figure 1:**
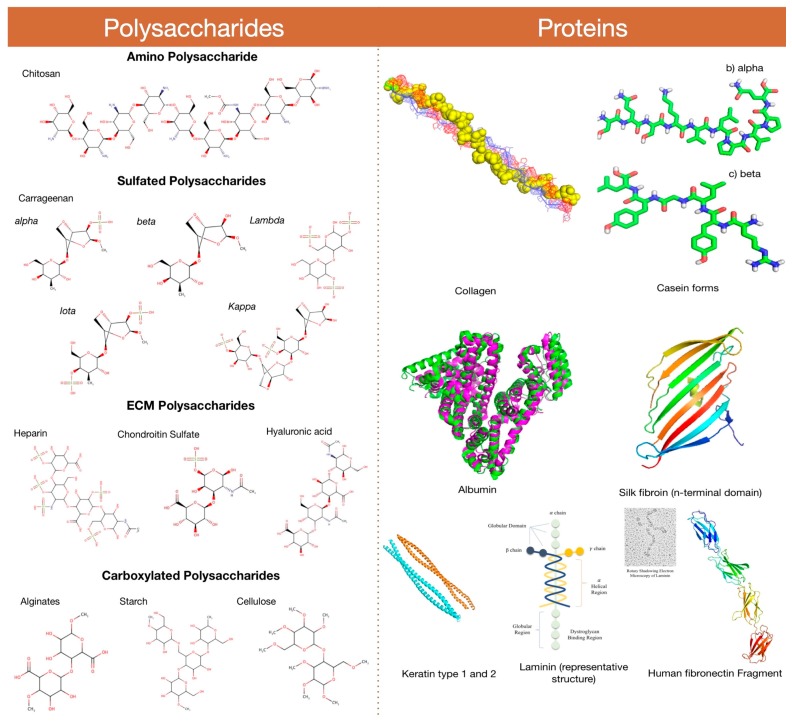
Structures of polysaccharides and proteins that are extensively explored for their uses in nanotechnology and regenerative medicine. Proteins show structural homology between human and bovine serum albumin structures (pink: human serum albumin and green: bovine serum albumin with RMSD score of 2.3). The figure also shows representative structure and electron microscope image of Laminin [8]. It also shows the human fibronectin fragment type III. Reproduced with permission from [8].

Natural polymers are those that are found in nature and extracted from sources like plants, animals (invertebrates and vertebrates) or microorganisms. They can be polysaccharides, proteins, or glycosaminoglycans (GAGs). Natural polymers exist either as structural or storage polymers, and many of them are extracted and used commercially for several foods, medical and industrial uses. For example, the extracellular matrix (ECM) is a non-cellular component of tissue or organ that provides a necessary microenvironment for performing various essential functions, such as providing physical and biochemical support for maintaining tissue morphology, cellular differentiation, and homeostasis [6]. In the last few years, ECMs belonging to a subclass of GAGs and fibrous proteins have been extensively explored for various nanomedicinal, tissue engineering, and wound healing applications. Most of the natural polymers are widely accepted in biomedical research and applications, as they are completely biodegradable, safe and non-immunogenic in nature [7]. Natural polymeric nanoconstructs are extensively identified as smart vectors and cellular support systems for therapeutic and wound healing processes, respectively. The present work focuses on carbohydrate- and protein-based natural polymers that are commercially explored as nanodepots and nanohealers (Figure 1). Mostly, these polymers are either utilized in native or chemically modified forms. Remarkably, natural polymers are preferred over synthetic polymers, due to their wide acceptance, abundance and cost effectiveness. Several commercially employed natural polymers in nanotechnology, medicine, and cellular regeneration technology are thoroughly discussed in the following sections.

## 2. Polysaccharides

### 2.1. Chitosan

Chitosan (CS) is a polysaccharide obtained by partial deacetylation of chitin isolated from the shells of crustaceans. Chemically, they are chains of sugars comprising subunits of glucosamine and *N*-acetyl glucosamine linked via β (1-4)-glycosidic linkages [7]. CS can be produced in several forms, like paste, powder, film, fiber etc., and is relatively reactive compared to chitin, which has been less frequently exploited due to its inertness. These amino-sugars have been well studied for their innumerable biomedical applications including tissue engineering, drug delivery, and scaffold synthesis. They are widely used for the synthesis of micro and nanoparticles for several biomedical applications [9]. Chemistry of the CS molecule plays an inevitable role in its wide usage in nanoparticle and microsphere preparation. The presence of the amino functional group imparts a positive charge and facilitates the binding of negatively charged polyelectrolytes, resulting in spontaneous nanoparticle formation. Cross-linking, desolvation with cationic salts, and polyelectrolyte complexation/ionic gelation—otherwise known as coacervation—are the major techniques employed for the fabrication of CS nanoparticles [10]. Many CS formulations are widely employed for drug delivery applications as a carrier for several drugs and small molecules. CS is a unique positively charged polysaccharide and it is broadly accepted for polyelectrolyte complexation with several other polysaccharides, which are polyanionic in nature. Recent advances have shown that CS-based polyelectrolyte complexes (PEC) have attracted nanotechnologists for fabricating nanoparticles, microcapsules, pellets, beads, tablets, gels, membranes, and films [11]. CS was first introduced as a drug carrier in 1989 for oral delivery application (one of the oldest biomaterial introduced for the field of nanomedicine) [12], followed by several other important uses (Table 2) [12,13,14,15,16,17].

#### 2.1.1. Nanotechnology Applications of CS and Its Composites

According to Kumbar et al., CS microspheres can be cross-linked using several methods comprising chemicals like glutaraldehyde and sulfuric acid, as well as via heat treatment for drug encapsulation and delivery [18]. CS nanoparticles have been synthesized in combination with several other polymers, and applied for spatial and temporal delivery of drugs and small molecules. Poly (lactic acid) (PLA)-CS nanoparticles were synthesized through emulsion method for the delivery of anti-HIV drug [19] and CS-dextran sulfate nanoparticles produced via coacervation were used for studying the encapsulation of anti-angiogenic peptide, arginine rich hexapeptide (ARH) [10]. Magnetic CS nanoparticles can be made using a tiny pool of water-in-oil immersion via microemulsion method [20]. This indicates the importance of CS in the field of nanodrug delivery.

CS aqueous solubility is pH dependent. It is basically insoluble in neutral and low pH regions; as a result, dilute acidic solvents such as acetic acid (1–3%, *v*/*v*) or citric acid (3–4%) are needed for making CS solutions. Interestingly, attempts to develop water-soluble derivatives of CS dates back to around the 1980s [21,22]. Derivatives of CS can be solubilized at neutral pH conditions, which helps in its biodegradation. For example, CS-PEC or *N*-acyl or *N*-alkyl derivatives of CS have shown improved water-soluble characteristics [23,24]. This has made Water-soluble CS derivatives (WCS) an attractive biomaterial for applications like wound healing and gene delivery [24,25]. Alkylated CS, sugar modified CS, quaternized CS, Copolymer grafted CS, natural polymer bound CS, oligomerized CS, PEGylated CS, and surfactant-attached CS are the different types of modified CS reported so far. Among these, *N*-trimethyl CS has been well studied for its brain targeting and anti-Alzheimer’s as well as neuroprotective drug delivery [26]. A series of CS derivatives were made by functionalization using carboxymethyl and sulfate groups to obtain carboxymethyl CS and CS sulfate respectively. 6-*O*-carboxymethylCS (6-O-CC), 2-*N* sulfated 6-*O*-carboxymethylCS (N-SOCC) and the 2-*N* and 3,6-*O* sulfated 6-*O*-carboxymethylCS (N, O-SOCC) were a few of the CS derivatives synthesized and tested for nanoparticle fabrication for efficient and highly protective delivery of basic fibroblast growth factor (bFGF). Among these, thiol-modified CS-sulfate (N, O-SOCC/CS) nanoparticles were found to be superior for the highly efficient loading and delivery of bFGF (basic fibroblast growth factor) due to their high affinity for bFGF [27]. Similarly, CS-*O*-isopropyl-5-*O*-d4T monophosphate conjugate nanoparticles were synthesized in combination with tripoly phosphate (TPP) and CS via ionotropic complexation to deliver anti-HIV polymeric drug under in vitro conditions. It was demonstrated that the cross-linked conjugate nanoparticles could show a mild sustained release of the coupled drug without leakage during circulation [28]. Similarly, Carboxymethyl CS was used to prepare magnetic nano-adsorbent by coating Fe_3_O_4_ nanoparticles for quenching polyvalent metal cations [29,30]. CS/sporopollenin microcapsules were synthesized as bio sorbents for binding and removal of heavy metals like Cd(II) and Zn(II) ions [31]. Furthermore, CS-based nanocarriers have been well studied and employed for gene delivery and siRNA delivery applications for controlling gene expressions [32]. Biocompatible multi-walled carbon nanotube (CNT)—CS- folate-based nanocomposites have been used for the delivery of green fluorescent protein. CNT-based delivery systems are efficient inorganic delivery systems that can be advantageously used for biomedical applications by controlling their length, size and functionalization. Toxicity was drastically reduced when CNT was functionalized with a CS-folate combination, and the transfection efficiency was also increased [33].

#### 2.1.2. CS Nanocomposites in Tissue Engineering and Regeneration

**Figure 2:**
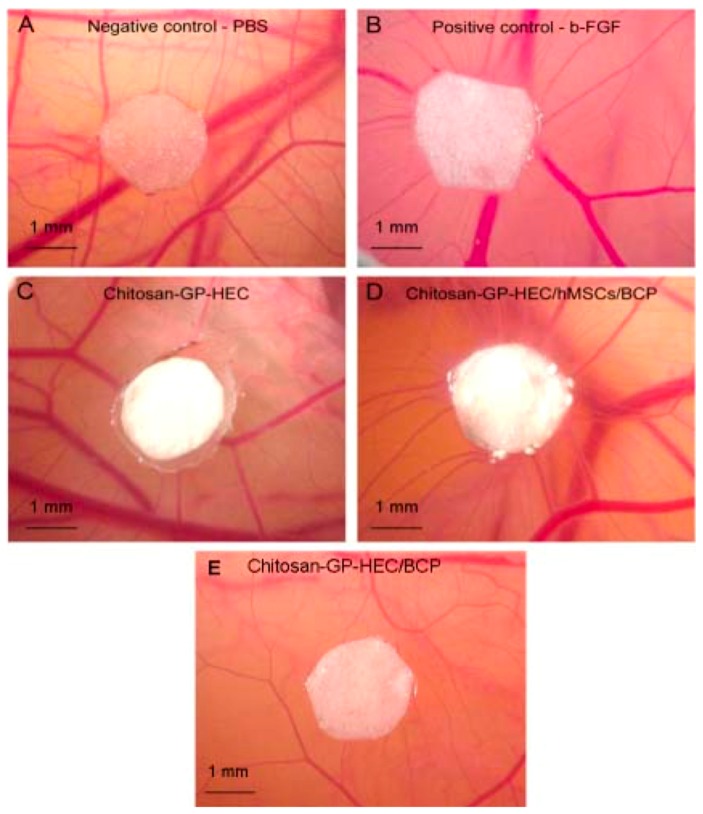
Extent of angiogenesis developed after three days of incubation with (**A**) PBS (negative control), (**B**) b-FGF (positive control), (**C**) CS-GP (glycerol phosphate)-HEC (hydroxyethyl propyl cellulose) hydrogel, (**D**) CS-GP-HEC/hMSCs (human derived mesenchymal stem cells)/BCP (biphasic calcium phosphate microparticles) hydrogel. Also known as with gel with hMSC. It clearly indicates formation of blood vessels and promotion of angiogenesis in hydrogel with hMSCs showing increased number of blood vessel formation compared to the control group (*p* < 0.05). and (**E**) CS GP-HEC/BCP hydrogel. Also known as gel without hMSCs. Reproduced with permission from [36].

Composite scaffolds of natural polymers are always promising for tissue engineering applications. They mimic the natural microenvironment of the extra cellular matrix and promote vascularization, tissue growth and regeneration. Nanosized hydroxyapatite/CS based tissue engineering scaffolds were produced via thermally induced phase separation and freeze drying and tested for in vitro and in vivo biological evaluation. Physical coexistence of hydroxyapatite nanoparticles with CS enhanced the mechanical strength and physical properties of the scaffold as compared to pure CS. Moreover, these scaffolds were shown to be good for growing mouse fibroblast cells in vitro [34]. In another work, Kuo et al. showed that a composite membrane comprising CaSO_4_/CS has the potential for guided tissue regeneration in rats. They fabricated two types of CaSO_4_/CS membranes from two different CS molecules with molecular weights of 70 kDa and 300 kDa, respectively, and tested for mechanical stability and clinical implications. It was observed that the CS containing membrane scaffolds was excellent in healing bone defects with good cell occlusion and inductive osteogenesis [35]. CS plays an important role in wound healing by modulating the function of inflammatory cells such as neutrophils, macrophages, fibroblasts and endothelial cells by promoting the formation and organization of granular tissue. The angiogenetic response is one of the prime responses for the tissue regeneration. A study showed that CS-glycerol phosphate (GP)-hydroxyethyl cellulose (HEC) hydrogel provided angiogenic property only presence of human mesenchymal stem cells [36]. However, native CS-HEC hydrogel does not exhibit any angiogenic effect. This study showed cytocompatibility, and the inert and non-inflammatory nature of the CS-HEC composite (as shown in Figure 2).

Perhaps nanofibers of CS-HEC with controlled delivery of angiogenic cytokines and vascular endothelial growth factor (VEGF) may aid by providing porous construct for helping tissue regeneration and angiogenesis. In contrast, in a negative report for the use of CS-HEC composite for angiogenesis, CS-collagen matrix was shown to improve the development of the vascular structure for endothelial cells. The study showed that even under in vivo conditions, collagen-CS matrix can stimulate increased vascular growth by recruiting Willebrand factor (WF+) and CXCR4+ endothelial/angiogenic cells. This shows that, although CS may not be angiogenic by itself, it may help in the growth of endothelial cells for development of improved vasculature [37]. Wound healing and tissue regeneration are often affected due to microbial infections. Hence, choice of biomaterials for regeneration medicine that exhibits anti-infective property becomes important. It is suggested that chitooligosaccharide may exhibit antimicrobial activity against *Actinobacillus actinomycetemcomitans* and *Streptococcus mutans* [38]. Moreover, water-soluble CS derivatives are more attractive for biomedical applications like tissue engineering, gene delivery, drug delivery, wound healing, permeation enhancement, etc. Quaternized CSs (QCSs) are highly promising in several such applications, as they are antimicrobial, biocompatible and bioadhesive in nature. QCSs have been thoroughly studied for their antimicrobial activity to kill free-swimming bacteria and to inhibit biofilm formation on clean surfaces. *N*, *N*, *N*-trimethyl CS (TMC) is a type of QCS, which was produced by reacting methyl iodide with CS at different degrees of quaternization. TMC was tested for its antimicrobial activity against Gram-positive bacteria *Staphylococcus epidermidis*, Gram-negative bacteria *Escherichia coli*, and yeast *Candida albicans*. Moreover, by eradicating the pre-attached bacterial and fungal cells from pre-existing biofilms, it was proved that they possess potent microbicidal activity [39]. A list of CS nanocomposites that were explored for their antibacterial activity is listed in Table 3 [40,41,42,43].

CS has been extensively explored for its use in soft tissue regeneration, for example, for the ocular or ophthalmic or corneal wound healing purposes. The cornea of the eye is a transparent barrier that prevents external damage to eyes, and helps in refracting light onto the retina. Most of the time, major corneal traumas are caused due to ulceration, bacterial or viral infections, and through heritable conditions. Damage to the cornea has led to vision loss in more than ten million people around the world. CS is one of the few natural biomaterials that has been extensively explored for corneal repair (as shown in Table 4). Previously, it was believed to be an inducer for vascular endothelial regeneration on topical application to rabbit’s eye [44]. Later, it was evaluated and conformed on a molecular scale for the mechanism of wound healing. It was found that CS helped in epithelial cell migration and proliferation in rabbit’s eye via. ERK pathway activation [45]. Moreover, a new CS derivative called CS-*N*-acetylcysteine (thiol derivative of CS also known as C-NAC) was introduced for corneal tissue regeneration. An in vivo study showed that CS-*N*-acetylcysteine containing eye drops improved corneal wound healing in a rabbit model [46]. Although CS has been potentially used in oculary drug delivery and wound healing, information about its use as nanofibrous scaffolds in corneal regeneration has been far overlooked.

### 2.2. Carrageenan

Carrageenans (CGs) are typical sulfated polysaccharides produced and extracted from some red algae belonging to *Gigartina*, *Chondrus Hypnea*, *Iridea* and *Eucheuma* species. These versatile hydrocolloids are widely accepted in the field of food, and have industrial applications such as acting as a stabilizing, gelling, thickening and suspending agent in water and milk systems. Applications of CGs are mostly exploited in the food industries. They have been a key ingredient in milk products, confectionaries, meat products, beverages, bakery products, dressing and soups for a decade. Similarly, they are used in cosmetics, toothpaste, emulsions, pet food, air fresheners, paints, etc. [47]. These linear polysaccharides have a high molecular weight with repeating units of sulfated and non-sulfated units of both galactose and 3,6-anhydrogalactose linked via alternating glycosidic linkages of β (1–3) and α (1–4) units. CGs are classified into three different types on the basis of the presence and number of sulfate groups in the galactose units. They are kappa (k), iota (*i*) and lambda (λ) CGs. The degree of sulfation is an important factor that can alter the physicochemical properties of CGs especially the gelling mechanism. Increased degree of sulfation will show decreased or low gel strength due to changes in gelling properties. Molecular weight, as well as the degree of sulfation, are the two major factors deciding the biological properties of CGs [48,49].

#### 2.2.1. Nanotechnology Applications of CG and Its Composites

In early 2000, k-CGs were used to develop nanocomplexes with metals hydroxides [50]. The use of CGs in nanodrug delivery was introduced nearly ten years ago with the purpose of increasing solubility and dissolution rate of hydrophobic molecules [51]. The study showed that hydrophilic λ-CGs, when used for development of hydrophobic molecule loaded CG NPs, increased the solubility of a molecule from <1 to 39 μg/mL. CG-CS hybrid nanoconstructs (100 to 1000 nm sizes) were also developed for the delivery of recombinant human erythropoietin [52]. This study showed the possibility of delivering macromolecules like proteins using CGs that could prove to be useful for the regenerative medicine.

Many biological properties and protective mechanisms against infectious agents have been reported for CGs to date. Antiviral properties are one of those properties that have predominantly been studied in the medical industry for inhibiting the growth and multiplication of various potent viruses using topical formulations. Grassauer et al. demonstrated that *i*-CGs are potent inhibitors of human rhinoviruses, which is a prevailing cause of the common cold [53]. These viruses are also implicated for increasing the severity of Chronic Obstructive Pulmonary Disease (COPD), asthma and are a cause for the loss of lung transplants. *i*-CGs inhibits the binding of virions to the primary human nasal epithelial cells, thereby preventing their multiplication and virus-induced cytopathic effect. However, clinical trials are required to prove its efficacy in the prophylaxis of human rhinovirus infections [53]. Another study revealed that λ-CGs could block the adsorption of feline herpes viruses (FHV) in the plaque assay. However, it neither showed a significant decrease in the virus titer nor reduced the clinical signs of FHV-1 conjunctivitis in the cat model. Nevertheless, CGs shortened the time period of positive virus isolation from the infected cat conjunctiva [54]. Despite significant research in antiviral studies using CGs, there are no ultimate formulations coming out to control viral infections like HIV. Carraguard is a kind of potential microbicide developed for testing HIV binding and inhibition of growth; it contains 3% PDR98- 15 k/λ CGs and has been successfully evaluated in clinical trials. These polyanionic molecules have the ability to prevent HIV-1 transmission by binding to the viral envelope. Moreover, CGs as microbicides could block the cell trafficking of macrophages from the vaginal compartment [55].

Similarly, CGs are well studied for their bionanotechnology applications for synthesizing nanoparticles and hydrogel formulations through polyelectrolyte complexation [56]. Metal binding and gelation properties of CGs were successfully employed for several bionano applications like reduction and stabilization of metal nanoparticles. Daniel-da-silva et al. used CGs as a colloidal stabilizer for making super paramagnetic nanoparticles with Fe_3_O_4_ via a co-precipitation method. CG-stabilized magnetite nanoparticles were small in size, and were capable of controlling chemical oxidation of Fe_3_O_4_ [57]. CGs form versatile hydrogels based on their degree of sulfation and polymerization. Many such types of gels have been studied for developing pH-responsive, salt-responsive and drug-responsive hydrogels. For example, an agarose and k-CG gel combination in an aqueous system was tested for competitive diffusion studies using dual drugs, showing a correlation between their adsorption isotherms [58]. A free radical polymerization technique was employed to prepare superabsorbent hydrogels, using a cross-linking graft copolymerized with acrylic acid and 2-acrylamide-2-methylpropanesulfonic acid onto k-CG. Various parameters were studied and optimized to obtain high swelling capacity and maximum water absorbency. Swelling ability was checked using multivalent cations and at different pH conditions. This superabsorbent gel was referred to as “anti-salt pH responsive superabsorbent hydrogel”, which could be an excellent candidate gel for drug delivery applications [59]. In another study, CG was used as efficient drug release modifier for ethylcellulose—coated pharmaceutical dosage forms. This study revealed that even a small amount of CG could effectively adjust the drug release kinetics and offer optimal therapeutic effects. Most importantly, CGs do not cause flocculation of the coated dispersion, and they were expected to impart long-term stability for storage upon proper curing [60]. CG was used to make thermal-sensitive composite gels with poloxamer-407 to enhance the sustained drug release of acyclovir in an in situ vaginal gel formulation. It was observed that under in vitro conditions, CG decreased the drug release profile and retarded the dissolution of poloxamer-407, which resulted in slow gel erosion. Whereas under in vivo conditions, CG significantly increased the local availability of the drug [61].

#### 2.2.2. CG Nanocomposites in Tissue Engineering and Regeneration

**Figure 3:**
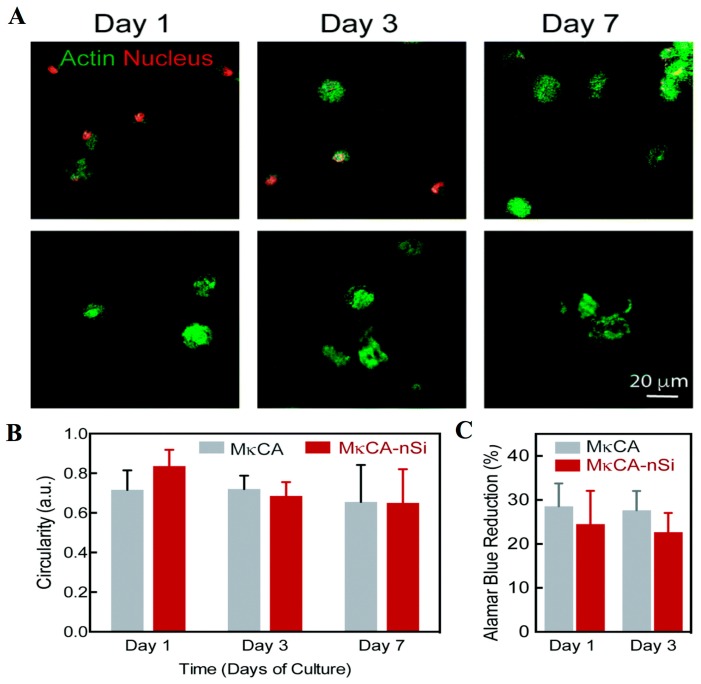
(**A**,**B**) Effect of nano silicate on spread ability of hMSCs over a period of 7 days. It can be seen from the confocal images that hMSC-treated cells were able to show circular morphology, indicating that hMSC can be delivered for cartilage regeneration. (**C**) Shows that there is no change in the metabolic activity of seeded cells. Reproduced with permission from [63].

Looking at the gel formation properties of CGs, it can be extensively explored for tissue engineering and regenerative medicine. Injectable biomaterial CG/nano-hydroxyapatite/collagen (nHAC/Carr) was developed as an injectable bone substitute [62]. Use of CGs for wound healing and tissue regeneration is in its nascence. Due to the better gelling nature of CG, it has been extensively explored for the development of nanogels for healing purposes. For example, recently, k-CG was used for the development of nanogels for the delivery of hMSCs (Figure 3) [63]. The hMSCs maintain their state in the G1/G0 phase of development. They maintain a circular morphology, which can be used cartilage tissue engineering. This suggests that perhaps a similar k-CGs nanoscaffold could be apt for tissue repair and wound healing purposes [64].

Furthermore, an antimicrobial-based approach for wound healing and tissue generation was also adopted for CG based formulations. Polyox-CG composite based solvent cast film consisting of streptomycin (30% *w*/*w*) and diclofenac (10% *w*/*w*) was developed for wound healing purposes. The films showed antimicrobial activity against *Staphylococcus aureus*, *Pseudomonas aeruginosa* and *E. coli* [65]. As discussed in the previous sections, being sulfated polysaccharides; CGs themselves possess protective mechanisms against infectious agents, which may aid in providing improved wound regeneration and healing.

### 2.3. Alginates

Alginates (AGs) are highly promising natural polymers used in biomedical applications and have been widely accepted as tissue engineering scaffolds. They possess excellent biological and physicochemical properties that ensure safe and nontoxic application in the medical industry. Biomaterials with typical gelling properties, biodegradability, biocompatibility, encapsulation properties and versatile geometric molding ability are always attractive for three-dimensional cell culture and tissue engineering [78]. AGs are such a biopolymer, and have been well studied in biotechnology and nanotechnology. These polysaccharides are arranged in a linear fashion, with β-d-mannuronic acid (M) and α-l-guluronic acid (G) residues bound via (1-4′) linkages. It is a homopolymer, and the monomers can exist in consecutive G/Consecutive M or alternate M and G residues, respectively. Mostly, AGs are extracted from several species of *Pheophycea* (Brown algae) including *Laminaria japonica*, *Laminaria digitata*, *Laminaria hyperborea*, *Ascophyllum nodosum*, and *Macrocystis pyrifera*. AGs are alkali-treated to obtain sodium AG, which is the commonly available form, and can be modified or blended together with various other polymers to obtain several composites [78].

#### 2.3.1. Nanotechnology Applications of AG and Its Composites

When sodium AG comes into contact with divalent cations, chains of poly-l-gluronic acid residues undergo folding to form the so-called “egg box” structure [79]. Calcium chloride solutions can induce gelation with AG to obtain calcium AG beads. Enteric coating polymers were used with varying degrees of coating on calcium AG beads (CABs) for creating sustained release formulations [80,81]. Sodium AG/poly-vinyl alcohol hydrogel beads were cross- linked with calcium ions and freeze thawing processes for enhancing the loading efficiency and swelling behavior [82]. In order to overcome the issues of entrapping hydrophilic drugs into CABs, various hydrophilic polymers like chondroitin sulfate, konjac glucomannan, sodium starch glycolate and xanthan gum were used to reinforce CABs for drug delivery applications. CABs loaded with intercalated complexes of propranolol HCl and magnesium aluminum silicate were used as microreservoirs for oral drug delivery system [79]. AGs have been widely employed as a carrier for sustained and controlled delivery of drugs, small bioactive molecules, proteins, and cells. Other than that, AGs are typically used for 3D cell culturing and tissue engineering. In a review by Venketesan et al., bone tissue engineering applications of AG composites with several modifications were explained in detail [78].

#### 2.3.2. AG Nanocomposites in Tissue Engineering and Regeneration

AG composites with various materials, like polymers, ceramics, metals, cells and growth factors were used to construct artificial bone that mimicked the natural functions of bone. Other natural polymers like chitin, CS, CGs and chondroitin sulfate have also been used for bone tissue engineering applications. Nevertheless, AGs have been well studied for their scaffold-forming applications to replace organs and tissues. Owing to their excellent biophysical properties and mechanical strength, AGs are actively used for regenerating skin, bone, pancreas, and liver. They are frequently modified to obtain hydrogels, microspheres, microcapsules, fibers etc. to study their tissue engineering ability. Among several techniques reported, electrospinning of AGs in combination with polymer solution is the best way to obtain nanofibrous scaffolds. AG scaffolds enhance bone tissue regeneration due to their natural similarity in biomechanical nature. For example, it provides a sufficient three-dimensional area, with highly interconnected pores for osteoblast and osteoclast differentiation. The mechanical strength offered is similar to that of the cortical bone, and the porosity ensures cell growth as well as vascularization, which help in cell migration as well [78]. However, the mechanical strength and cell adhesive qualities of AG scaffolds can be increased by the addition of materials like bioglass, hydroxyapatite, calcium phosphate cement, natural and synthetic polymers etc. Similarly, the gelation rate can be controlled as a factor in order to fabricate gels appropriate for various applications by using a crosslinking agent or a crosslinking retarding agent. CaSO_4_ has been used as a crosslinking agent, and Na_2_HPO_4_ as a cross-linking retarding agent to control the rate of gelation. PVA-blended AG injectable hydrogels have been developed and tested for in vitro chondrocyte growth by controlling the rate of gelation [83]. In a study by Huang et al., it was shown that microarray patterns of CAG can be made on the desired location using electrodeposition on a photoconductive electrode system. Micro patterning of cell-encapsulated CAG is a simple, rapid and flexible method for creating cellular arrays based on AG using light-addressable electrodeposition [84].

AG is typically used for 3D cell culturing and tissue engineering. Due to its good water solubility profile, AG is commonly used in a composite state with a variety of natural and synthetic biomaterials for the development of formulations for wound healing and tissue regeneration purposes (shown in Figure 1). An AG-CS scaffold was able to support undifferentiated mesenchymal stem cells (MSCs) for 14 days in vitro, and promoted a spherical morphology. In vivo evaluation of the scaffold also showed partial defect closure after 16 days in the treatment group [85]. Further, AG-collagen and AG-gelatin hydrogels were developed in order to aid wound healing under in vivo conditions [86]. A study showed that at a cellular level, AG fibrous and microsphere scaffolds impede the wound healing process and cause an inflammatory response compared to hydrogel [87,88]. Hence, there are two basic ways by which it is possible to avoid the inflammatory response generated by sodium AG: (a) developing nanogels of native AG; and (b) developing nanofibers from an AG-polymer composite. Recently, histological evaluation was conducted of ciprofloxacin-loaded AG-PVA nanofibers developed as a wound dressing material. In comparison to commercial wound dressing materials, the nanofibrous mat was able to provide better epithelialization, epidermis characteristics, vascularization and formation of hair follicles. This study showed that the AG-PVA mat is appropriate as an artificial skin scaffold for aiding tissue regeneration and wound healing [89].

**Figure 4:**
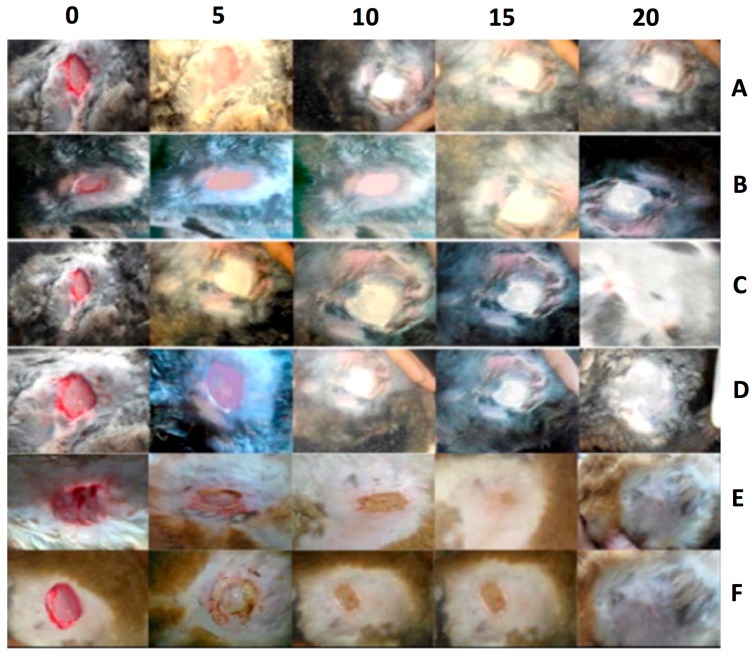
Wound healing using nanofiber patch healing with AG-PVA fibrous mat over a period of 20 days in comparison to control group. (**A**) control untreated group; (**B**) PVA nanofibers; (**C**) native AG-PVA nanofibers; (**D**) drug-loaded PVA fibers; (**E**) drug-loaded AG-PVA nanofibers; (**F**) marketed standard formulation. The study clearly indicates that drug-loaded AG-PVA nanofibers provided relatively better wound healing properties. Reproduced with permission from [90].

Along similar lines, an AG-PVA nanofiber mat was developed for the delivery of antibiotics (ciprofloxacin) to the wound site for aiding the healing process (as shown in Figure 4). The fibrous mat aided in production of hydroxyproline at the wound site, but was higher in the case of AG-PVA mat, indicating a relatively higher wound healing property [90]. Moreover, the images also show that the AG-PVA native wound healing patch also shows good wound healing properties compared to the negative control and PVA nanofibers. This may be attributed to their impeding the inflammatory response, as shown by the hydrogel-based formulations. These studies clearly indicate the importance of AG-polymer blends for wound healing purposes. It also shows that the native polysaccharide AG may only be useful when used in nanohydrogel formulations for tissue regeneration or healing applications.

In addition, structurally modified calcium AG hydrogels were also shown to have improved ocular wound healing properties (as shown in Table 4). These modified hydrogels can also be used for the transport and storage of the corneal epithelial cells [68]. Moreover, a hybrid composite hydrogel of CS and oxidized sodium AG was developed for the reconstruction of the corneal endothelium. The study showed that the gel could be degraded within 30 days with little inflammatory response. The in vivo experiments showed that after 30 days (following implanting the gel), the eyeballs of the treated group looked almost the same as the control group. The study showed that the composite biomaterial studied was safe for the encapsulation of corneal endothelial cells. This provides an opportunity for the reconstruction of the corneal endothelium, as well [91].

### 2.4. Starch

Starch is the major storage polysaccharide present in plants, and it is the main energy-giving form of carbohydrates in the human diet. Starch can be extracted from several food sources, like rice, corn, potato, wheat, cassava etc. Chemically, starch is a polysaccharide with two different polymers coexisting naturally as linear and branched, with amylose and amylopectin, respectively. Both of them consist of repeating units of d-glucose monomers connected either through α (1-4) linkage for linear, amylose and α (1-6) linkage for branched amylopectin polymer chains. Based on their physicochemical and biological properties, they have also been used for several bionano applications apart from their food and industrial uses.

#### 2.4.1. Nanotechnology Applications of Starch and Its Composites

Starch molecules are used in eco-friendly polymer production by doping the synthetic polymers like polypropylene carbonate, fabrication of anisotropic transparent gels, starch granules, nanostructures for protein and drug delivery, etc. [92]. Moreover, starch derivatives can be prepared by modifying starch molecules via physical, chemical or enzymatic processes, thereby enhancing the materialistic and biological properties. Starch nanoparticles or microparticles can be prepared using several techniques, such as precipitation, solvent evaporation, spray drying, emulsion cross-linking, etc. Cross-linked starch microparticles are excellent in their stability for storage and swelling, biocompatibility, high temperature, high shear rate and biodegradability [93,94]. Nanoparticles made using taro starch hydrolysis were reinforced in cornstarch film for enhancing their mechanical properties, temperature stability and structural features [95,96,97]. The major reason for the application of starch nanocrystals to reinforce several polymers and films is mainly due to the semi-crystalline nature of the starch granules. This abundant renewable polysaccharide is semi-crystalline, with alternate amorphous and crystalline domains; amorphous domains can be easily removed by acid hydrolysis, ensuring starch nanocrystals. High performance and highly durable materials can be fabricated by reinforcing starch nanocrystals [98]. Similarly, functionalization or chemical modification of starch using cationic molecules will facilitate polyelectrolyte complexation of cationic starch molecules with anionic surfactants to give rise to starch nanoparticles [99]. Apart from that, starch has been widely used for reduction and stabilization of metal oxides to produce metal nanoparticles [100]. Just like various other polymer nanoparticles, cross-linked starch nanoparticles can be used for controlled and sustained drug delivery [101,102]. Cross-linked starch nanoparticles have great potential for controlled drug delivery, and *N*, *N*-bisacryloylcrystamine cross-linked nanoparticles were demonstrated to have reduction-sensitive properties with controlled drug release [103]. Starch-based hydrogels have also been used for drug release studies by cross-linking and addition of functionalizing moieties. Li et al. showed an in-situ hydrogel preparation through Schiff reaction using starch nanoparticles and polyvinylamine by cross-linking and encapsulating the cancer drug, doxorubicin [104].

#### 2.4.2. Starch in Tissue Engineering and Regeneration

Like other polysaccharides, starch is another biomaterial that is extensively applied for tissue engineering and regeneration applications by the formation of macro structures. Albino et al. developed PEC of polycaprolactone and starch-based fibrous scaffolds for bone tissue engineering applications. The nanofiber mesh was developed with size range 400 nm to 1.4 μm. The mesh was able to support efficient growth of human osteoblast cells, and efficiently promoted synthesis DNA indicating efficient bone tissue development [105]. Recently, porous starch/cellulose nanofiber composites were used for tissue engineering applications. The size of the nanofibers was found to be 40 to 90 nm [106]. Starch has also been used for the development of 3D nanofibrous composite scaffolds, which can be used for bone tissue engineering, cellular regeneration, and growth. Apart from the composite blends, starch is chemically modified to achieve the desired degree of wound healing and tissue regeneration properties. The most commonly used chemically modified forms include: (a) cadexomer, (b) oxidized starch (OS), and (c) hydroxyethyl starch (HES). Cadexomer is a hydrophilic starch crosslinked with epichlorohydrin [107]. It is popularly incorporated with 0.9% (*w*/*w*) iodine to form cadexomer-iodine (CI) ointment for wound healing. Here, cadexomer serves as a carrier for iodine [108]. It swells when applied to the wounds and releases the iodine slowly into them, providing antiseptic properties. Like iodine, cadexomer can also be used as a carrier for other growth factors that can aid rapid wound healing.

**Figure 5:**
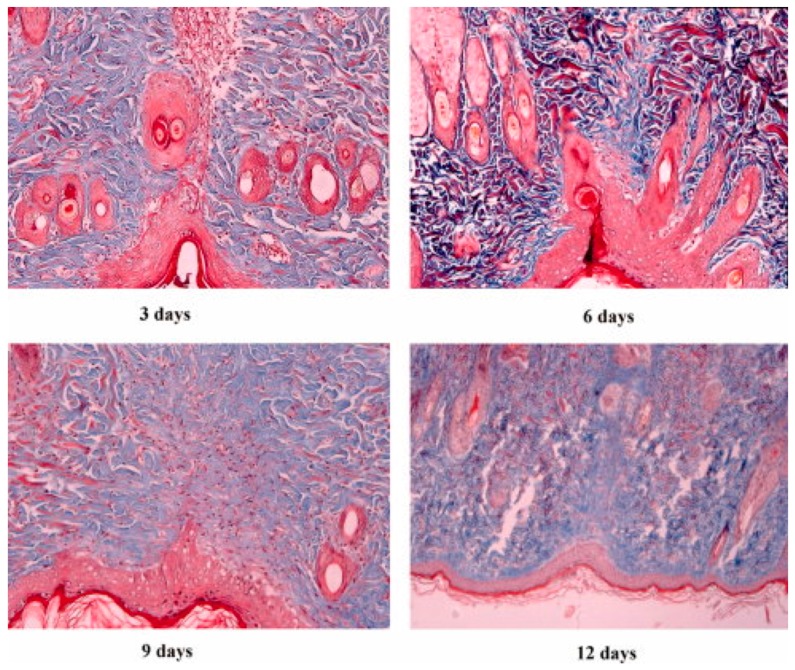
Cross section of porcine skin glued with HES-gelatin glue. It can be seen that by the end of 12th day the glue had completely disintegrated, and new connective tissue had developed. Reproduced with permission from [111].

OS is synthesized by chemically treating starch with oxidizing reagents. It is an excellent material known for its film-forming capability, due to low viscosity and high stability [109]. Being a negatively charged molecule it can easily form PEC with oppositely charged polymers. CS-OS nanofiber-based scaffolds were developed for bone tissue regeneration. A study with MG63 (osteoblast cells) showed that an increased quantity of starch was able to improve cell viability [110]. OS-PVA (poly-vinyl alcohol) blends have also been used for the development of nanofibers for tissue regeneration and wound healing purposes. HES is obtained through hydrolysis, followed by hydroxyethylation, of amylopectin. It can also be produced by exposure of starch to ethylene oxide at <50 °C. It is most commonly used as a tissue adhesive for the closure of wounds [111]. It has been extensively explored for its potential for wound closure under in vivo conditions. Histopathology studies clearly showed that gelatin (gel)-HES and gel-dextran were found to have higher binding and adhesive strength on porcine skin compared to CS-dextran and fibrin glues (Figure 5). These studies clearly indicate the cytocompatibility of HES-based composites for wound healing purposes. Apart from being used as a gel that works as glue for wound closure, HES has also been explored for its nanodrug delivery application. Although, whether it can be used for the development of 3D nanofiber-based scaffolds for tissue regeneration and wound healing remains unclear.

Moreover, there is no evidence to suggest that native or chemically modified starch possesses antimicrobial or anti-infective properties. Although, this biomaterial has been extensively explored for the development of NP or polymeric blends with starch to achieve antimicrobial properties. Starch-CS composite film was prepared with volatile oils to show antibacterial activities against *Salmonella anatum*, *Staphylococcus aureus*, *Bacillus cereus* and *Aspergillus niger* [112,113,114]. Starch PEC blends with synthetic and natural polymers have been extensively used for wound healing purposes [109,115]. These studies clearly indicate that, apart from the formation of films and hydrogel, starch (in both its native and chemically modified forms) can be used for the formation of nanofibers by forming nanocomposites. Despite being an interesting biomaterial for drug delivery purposes, use of starch nanofibers or nanogels for wound healing and regeneration purposes remains unexplored territory [116]. Looking at the success of starch biomaterial dressings, starch-based nanoconstructs in regenerative medicine would be a promising field to consider.

### 2.5. Cellulose

Consumer industry prefers products that are more biocompatible and biodegradable, especially those that have low human/animal/environmental risk factors and those based on carbon-neutral non-petroleum products. After several types of research, plant nanotechnologists narrowed down their research to the base fundamental reinforcement unit of trees and plants, cellulose nanocrystals, in order to incorporate them in various materials for enhancing mechanical properties [117]. Cellulose is a structural polysaccharide mainly extracted from the rigid cell walls of plants. They form linear polysaccharide chains with beta acetal-linked glucose units as monomers. Unlike starch, cellulose is indigestible by the human GI system due to its beta acetal linkages, and hence is not used in human diet. Fortunately, we found it highly promising for innumerable industrial applications, including paper products, cloth material like cotton, linen, and rayon, cellulose acetate films, nanocrystals and nanowhiskers for reinforcement of films and scaffolds, etc.

#### 2.5.1. Nanotechnology Applications of Cellulose and Its Composites

Cellulose can impart good mechanical strength and thermal stability to fibers and films. Cellulose nanoparticles are ideal for reinforcement of materials due to their high aspect ratio, low density and the presence of a reactive hydroxyl functional group. Surface functionalization facilitates self-assembly and controlled dispersion of cellulose nanoparticles with a variety of polymers and surfactants. Based on the chemistry and material properties, cellulose nanocrystals have a wide range of applications in the biomedical and nanotechnology industries like barrier films, transparent films, reinforcing materials for polymer matrices, implants in biomedical applications, drug delivery, pharmaceuticals, separation membranes, etc. [117]. Cellulose hydrogels can be prepared by cross-linking in sodium hydroxide/urea aqueous solvent media and it was shown to have a higher water uptake capacity of 99.6%. These cellulose hydrogels were die-cast and evaporated to fabricate 3D-ordered cellulose films. The stacked cellulose sheets endowed high tensile strength and flexibility to the cellulose 3D film [118]. Recently, recycled cellulose-based aerogels were prepared from paper waste for absorbing oils, and exhibited a very stable super-hydrophobicity. It was shown to be a promising sorbent for cleaning oil spills [119]. The structural, mechanical and sensorial characteristics of cellulose nanocrystals loaded in cold-set protein gel were compared with ordinary protein gels. The protein gel characteristics were able be modified on impregnation of the cellulose with micro- and nanocrystals. Cellulose on acid hydrolysis yields crystalline cellulose after complete denaturation of its amorphous parts. These crystalline moieties can be used for the reinforcement of a variety of materials to enhance their functional characteristics. Cellulose nanowhiskers obtained after sulfuric acid hydrolysis were grafted with amino group containing Polyamidoamine (PAMAM) dendrimers. Cellulose nanowhiskers are considered to be a green nanomaterial with remarkable mechanical and chemical properties [120]. Cellulose nanospicules from nanofibers and nanorods from nanofilms were prepared by electrospinning and hydrolysis/hydrogenation from one-pot synthesis. The major advantage of this method is the production of low molecular weight polysaccharides via depolymerization and, in turn, production of biofuel, energy and several useful chemicals from renewable biomass [121]. Hence, cellulose nanoparticles gained much interest in nanotechnology and biomedical industry as a versatile nanofiller material.

#### 2.5.2. Cellulose Nanoconstructs in Tissue Engineering and Regeneration

Cellulose nanoparticles are ideal for reinforcement of materials due to their high aspect ratio, low density and the presence of a reactive hydroxyl functional group. Also, cellulose hydrogels can be prepared by cross-linking in sodium hydroxide/urea aqueous solvent media, and this has been shown to result in a higher water uptake capacity of 99.6%. These cellulose hydrogels were die-cast and evaporated in order to fabricate 3D-ordered cellulose films. The stacked cellulose sheets endow the cellulose 3D film with high tensile strength and flexibility [73]. Cellulose is also used for the preparation of scaffolds that can be used for tissue engineering, regeneration or wound healing purposes.

**Figure 6:**
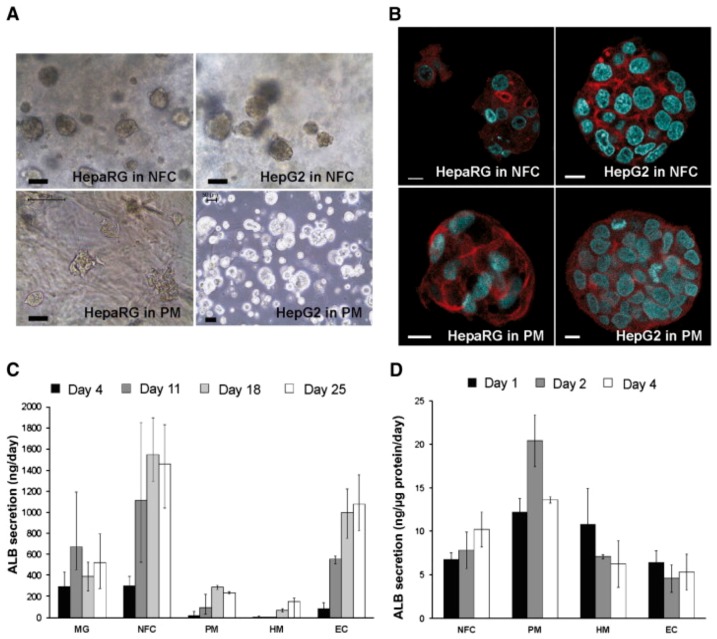
(**A**) Circular morphology of the HepaRG and HepG2 cells in nanofibrillar cellulose (NFC) in comparison to the Puramatrix™ (PM) standard. Both showed similar cellular morphologies. (**B**) Shows the confocal images of the cells with structural staining of filamentous actin (red) and nuclei (blue). It can be seen that, in contrast to HepG2 cells, filamentous actin shows accumulation at the site of the apical membrane of HepaRG cells. This may be due to the in vivo-like polarity, which is known to be associated with canaliculus formation. The differentiation state of the HepaRG cells was also confirmed using secretion of albumin as a marker. (**C**) Shows the secretion of albumin by HepaRG. (**D**) Shows the secretion of albumin from HepG2 cells. Both the studies clearly indicate a release of albumin in cells treated with NFC that is comparable to standard commercial formulation. Reproduced with permission from [123].

Cellulose biomaterial has been extensively tested and utilized for tissue reconstruction for vascular tissue engineering, and bone tissue reconstruction; it is also used for the engineering of skeletal muscle, heart valves, cardiac muscle and other things. Nanofiber has also been extensively utilized for carrying liver cells and islets of Langerhans [122,123]. A study in which nanofibrillar cellulose hydrogel was used for the 3D culture of liver cells (HepG2 and HepaRG) showed the formation of multicellular spheroids (shown in Figure 6) in nanofibrilar cellulose (NFC) hydrogel; a similar trend was observed in Extracel™, HydroMatrix™ and PuraMatrixTM (data was not shown for these formulations).

Cellulose has also been successful as a surgical implant for guided tissue regeneration [122]. Recently, improvement of porosity has been a prime focus of attention for the development of porous cellulose-based nanoconstructs. A 3D porous nanocomposite scaffold of cellulose nanofiber was developed for cartilage tissue engineering [124]. These studies clearly indicate the cytocompatibility, cellular adhesion and proliferation properties of cellulose. An in vivo study on rectus abdominis muscle defects in mice showed that cellulose biomaterial has a relatively better wound healing ability, compared to gelatin materials [125]. Wood-based nanofibril cellulose (NFC) was developed recently as a novel wound healing dressing material. The study showed that NFC has a relatively faster healing property, compared to Suprathel^®^ [126].

There is no direct assay that can suggest the possible mode of action of wound healing for cellulose. Moreover, cellulose as such does not have any antimicrobial activity. However, it was shown that oxidized regenerated cellulose (ORC) could potentially inactivate harmful proteases, oxygen free radicals and excess metal ions in chronic wound fluid [127]. Perhaps a similar function is played by cellulose nanoconstructs for tissue regeneration/wound healing. Furthermore, cellulose acetate and polyester urethane (PEU) blends were used for the development of nanofiber membranes loaded with polyhexamethylene biguanide (PHMB). The study showed that the nanofibers not only worked as drug delivery vectors, but also helped in wound healing, due to anti-microbial activity exhibited by PHMB [128].

Moreover, in the late 1990s, methylated derivatives of cellulose (methyl cellulose (MC) and carboxy methyl cellulose (CMC)) were shown to have corneal wound healing properties [129]. In a study, CMC was used in an artificial tear formulation in order to understand the mechanism of corneal wound healing. It was shown that the artificial tears facilitated interaction with corneal epithelial cells (HCECs), and helped in the healing process. The study showed that CMC remained bound to HCECs for 2 h, and helped in the re-epithelialization of HCECs scratched in in vitro and in vivo rabbit corneal wounds. It was shown that the interaction of CMC-mediated healing probably occurred due to interaction of glucopyranose subunits with glucose transporters of cells [130]. Recently, carboxylated nanowhiskers of cellulose were used for the formation of nano hydrogel for ophthalmic applications. The hydrogel showed excellent biocompatibility with corneal epithelial cells [70]. However, the use of this formulation for wound healing purposes is unknown. Apparently, a molecular understanding of the use of the cellulose derivatives for ocular therapy is a relatively new domain. As a result, there is limited information about the use of cellulose-based nano scaffolds for ocular wound healing.

These studies clearly indicate the importance of cellulose in the formation of fibers and its use in wound healing applications. Although various natural polysaccharides have been extensively explored as nanohealers, biomolecules like proteins also play an important role in performing various cellular functions like growth, migration, and development.

### 2.6. Heparin

Glycosaminoglycans (GAG) are a class of compounds that constitute long unbranched polysaccharide chains with repeating disaccharide units. Heparin (HP) is a GAG that contains repeating disaccharide units of 1,4-linked d-glucuronic acid or l-iduronic acid and glucosamine residues. It is a highly sulfated polysaccharide with sulfation at different positions within the constitutive residues. Apart from the sulfate group, HP contains a carboxylic acid group, making it the highest negatively charged biomolecule ever known. This highly sulfated GAG is mostly used as an anticoagulant and, moreover, its antiviral, anti-proliferative, antitumor and immunomodulating properties are also well established [48]. HP sulfate is a structurally similar GAG to HP, which has exceptional biological properties, such as cell adhesion, cellular growth, cellular proliferation, cellular binding, and inhibition of cancer angiogenesis, viral invasion, and tumor metastases [131]. Besides these, high negative charge density and versatile physicochemical properties make HP and its derivatives promising candidates in bionano and biomedical applications.

#### 2.6.1. Nanotechnology Applications of HP and Its Composites

In a recent review by Yang et al., various bionano applications of HP-based nano carriers were summarized, and their unique advantages in cancer nanotechnology were discussed [132]. Several combinations that have HP as a sole constituent polymer in a nanocomposite formulation have been studied for delivery of drugs, small molecules, peptides, proteins, siRNA, etc. In particular, they have been used for the synthesis of polymeric nanoparticles, drug conjugates, nanogels, polyelectrolyte complex nanoparticles, as well as for stabilizing or coating nanocrystals and inorganic nanoparticles. Superficial coating of HP onto certain substrates and nanoparticles can potentially suppress the intervention of the immune system by making it biocompatible. This probably occurs by preventing the opsonization and escape from complement activation cascade of HP-coated nanocarriers, helping it to remain in the blood circulation for a longer time [133]. However, application of low molecular weight HPs (LMWHP) is preferable to unfractionated HP, due to the occurrence of hemorrhagic complications like thrombocytopenia. LMWHPs have been found to better in its offer potential benefits over unfractionated HPs in terms of their anti-inflammatory, anticoagulant, anti-metastatic and antitumor abilities [134]. Generally, LMWHPs are administered parenterally as standard anticoagulants for patients undergoing abdominal surgery and arthroplasty to prevent deep vein thrombosis. In order to develop an oral formulation, tinzaparin nanoparticles were prepared with a combination of polyester and a polycationic methacrylate and given orally to fasted rabbits. The results of this experiment suggest that, on encapsulation of LMWHP, tinzaparin into nanoparticles could contribute to oral efficiency with an anticoagulant effect that can be sustained for a longer duration [135]. In a similar experiment, polymeric nanoparticles prepared using polycaprolactone (PCL) and poly (lactic-*co*-glycolic acid) (PLGA), along with positively charged polymers like Eutragit RS and RL, were also studied for the bioavailability and efficiency of HP when administered orally to rabbits [136]. Likewise, LMWH and protamine nanoparticles were successfully used for encapsulation and delivery of HP-binding growth factors like fibroblast growth factor. Growth factor molecules were found to be protected inside LMWHP/protamine nanoparticles, even after heat inactivation and proteolysis, indicating the effectiveness of HP nanocarriers in protein delivery [137]. Water-soluble HP-paclitaxel conjugates were tested for cancer targeting, thereby evaluating the efficiency of delivering drug to solid tumors using HP-based nanocarriers [138]. Experiments show that certain modifications or functionalizations of HP with appropriate ligands can increase the cellular uptake through site-specific targeting of cancers. In vivo studies demonstrate the effectiveness of tumor drug delivery and reduction of toxicity using an LMWHP conjugated with all-trans-retinoid acid cofactors [139]. HP conjugated with folate and folate-PEG complexes have each been used for the delivery of antineoplastic drug paclitaxel to cancer cells positive for folate receptors [140]. In the same way, inorganic/organic nanoparticles were prepared for drug delivery applications by co-precipitating Ca^2+^ ions with carbonate and phosphate ions in the presence of HP [141]. Dendronized HP is another variant of an HP-based nanocarrier used for drug delivery and cancer therapy. Nanosized dendrimers are always promising for the delivery of several payloads due to their monodisperse size, water solubility, multivalency, and surface functionalizing properties. Doxorubicin conjugated to dendronized-HP via hydrazone linkage has been successfully evaluated for tumor inhibition in vivo [142]. As an alternative to several polymeric nanoparticles, inorganic/metal nanoparticles and nanocrystals have been widely used for tumor inhibition based on photothermal and photodynamic therapy. Li et al. developed an HP-Au nanoparticle complex conjugated with a photosensitizer, pheophorbide (PhA), for photodynamic therapy of cancers. These hybrid nanoparticles showed glutathione-mediated switchable photoactivity by observable quenching and dequenching of fluorescence. Remarkable phototoxicity was observed in a GSH-rich medium with production of singlet oxygen on illumination. Thus, a PhA-HP-Au nanocomplex was considered efficient for photodynamic therapy of tumors in mice with prolonged circulation and target-specific characteristics [143]. Another example of HP-bound inorganic nanoparticles is nanoceria, which are cerium oxide nanoparticles synthesized via a spray pyrolysis technique and functionalized with HP molecules. HP-functionalized nanoceria hold superior biological properties with increased cellular uptake and reactive oxygen species (ROS) scavenging [144]. Apart from protein and drug delivery applications, tissue engineering and cell culturing applications have also been thoroughly studied using HP-coated polymeric substrates and scaffolds. HP/poly-(l-lysine) nanoparticles were adsorbed onto the surface of PLGA microspheres to demonstrate the cell adhesion and growth [145]. In general, HP molecules have been widely applied for decades to reduce the thrombogenicity of materials in contact with blood. Taking this property into consideration along with several other biological and physicochemical properties, HP molecules have been widely used as a biologically active polymer for biotechnology and nanotechnology applications [146,147].

#### 2.6.2. HP Nanoconstructs in Tissue Engineering and Regeneration

**Figure 7:**
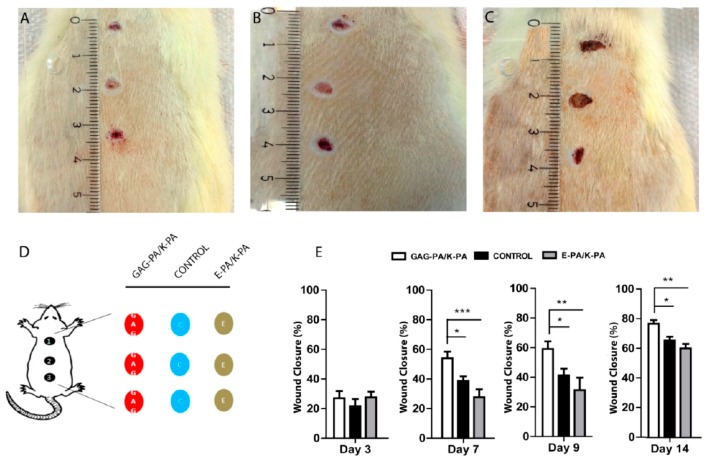
Wound closure in diabetic rats for subjects treated with (**A**) GAG (glycosaminoglycan)-PA (Peptide amphiphiles)/K-PA (also known as positively charged heparin mimetic PAs), (**B**) PBS control, and (**C**) E-PA (negatively charged heparin mimetic PA)/K-PA. (**D**) shows the schematic diagram for the location of wound on the rats that were treated with GAG-PA/K-PA, PBS and E-PA/K-PA samples. (**E**) Shows the % wound closure studied for 14 days after the treatment. It can be seen that after 14 days of the treatment compared to control. It can be seen that GAG-PA/K-PA bio gel-treated group provided significant wound healing property on 14th day of treatment, compared to the E-PA/K-PA- or PBS-treated control groups (one way ANOVA test with * *p* < 0.05, ** *p* < 0.01, *** *p* < 0.001). Reproduced with permission from [150].

HP can be easily incorporated in nanoconstructs that can be easily be recognized by the HP-binding domains of proteins (fibroblast growth factors), which helps in cell proliferation and osteogenic cell differentiation (bone morphogenic proteins, pleiotrophin). It was shown that HP loaded with VEGF nanofibers exhibited better angiogenic growth than VEGF alone [147,148]. Moreover, HP mimetic nanofibers that mimicked the chemistry of HP sulfate and binds to VEGF helps in the angiogenesis. Mammadov et al. showed that the HP mimetic nanofiber scaffold binds with greater affinity to hepatocyte growth factor (HGF) and fibroblast growth factor-2 (FGF-2) [149]. The study found the HP binding domain on VEGF and this was found to be critical for nanofibers. Recently, HP mimetic nanofibers were also tested as bioactive gels for wound healing purposes in STZ-induced diabetic rats (as shown in Figure 7) [150].

HP-coated aligned nanofiber scaffolds were also found to be helpful in increasing endothelial cell infiltration and dermal tissue remodeling [151,152]. Similarly, PCL-HP hybrid scaffolds have shown controlled release of VEGF and improved angiogenic properties. An electrospun PCL scaffold immobilized with basic fibroblast growth factor (bFGF) has also been developed. The study showed sustained delivery of bFGF under both in vitro and in vivo conditions. Several hybrid nanofibers, like gelatin-HP, HP-PCL and others, were developed to have improved tissue engineering and wound healing purposes [153].

Heparin has also been evaluated for its significance in corneal wound healing applications. Heparin has a ligand binding site on Epidermal growth factor receptor (EGF) to form HP-EGF complex for carrying increased epithelial wound healing process. It has also been suggested that HP-EGF knockout mice die soon after birth. It is interesting to note that the study fortifies the use of HP in the healing of corneal epithelium compared to EGF [72]. Perhaps the nanofibrous scaffold that has been developed for epidermal wound healing applications may also be applied for corneal wound healing applications in the future.

### 2.7. Hyaluronic Acid

Hyaluronic acid (HA) is a typical ECM polymer that has been widely employed for several biomedical and nanotechnological applications. In our previous review work, pharmaceutically versatile sulfated polysaccharides, including various ECM polysaccharides, were studied [48]. Many material scientists have published expert reviews on the relevance and significance of HA as a biomaterial in nanomedicine [154,155,156]. Being an ECM, HA plays an important role in wound healing and tissue regeneration. During wound healing, the concentration of HA is usually increased, which is contributed to initially by platelets and later by the damaged endothelial cells. It has been observed that HA in its native state helps in improving the wound healing process. HA is produced as (a) high molecular weight HA (HW-HA), and (b) low molecular weight HA (LW-HA). During the wound healing process, the HA secreted by platelets and endothelial cells undergoes breakdown into smaller fragments by hyaluronidase enzyme. These smaller fragments during days 3 to 5 of wound healing help in inducing mitosis, sprouting, tropism of the endothelial cells, and the provision of angiogenesis and neovascularization. Moreover, expression of vascular endothelial growth factor is also controlled by HA. During day 6, migration and proliferation of fibroblast is also governed by HA. It also stimulates synthesis of collagen type III by fibroblasts and other ECM components that can help in tissue recovery. Lastly, HA, along with CD44 in a complex biochemical process, regulates keratinocyte proliferation and migration during re-epithelization [157].

With regard to the biochemical significance of HA in tissue repair, it has been proposed as an interesting biomaterial for nanomedicine and cellular regeneration. Uppal et al. developed an HA nanofiber-based wound dressing formulation. The preclinical testing of these HA nanofibers on pig wounds showed that they were able to provide excellent wound healing on day 10, compared to native solid HA biomaterial. HA nanofibers provide the required air permeation at the wound site, better than native solid HA, which aids in the cellular migration and proliferation to promote the growth of tissue and faster wound healing [158]. With regard to the significance of HA in wound healing, the material was explored in combination with PLGA, PCL, PEO, CS [159,160,161]. HA nanofibers have also been explored for anti-infective properties. PEO-HA nanofibers have been developed for the delivery of kanamycin for anti-*Listeria monocytogenes* [160]. Further, thiolated (SH)-HA nanofibers have been developed for the delivery of tenofovir (TFV) for anti-HIV activity. These nanofibers are seminal enzyme bio responsive and mucoadhesive in nature. They release drug under the influence of seminal hyaluronidase after 1 h of exposure [162]. These examples clearly indicate that, apart from being a natural wound healing vehicle, HA can also be used for the delivery of antibiotics that further aid in anti-infective mediated wound healing properties. In addition, HA is also applied to the eye as a lubricant for the treatment of dry eye syndrome. However, it gets cleared out quickly due to limited cellular adhesion. As a result, Lee et al. developed HA-loaded polymer-peptide conjugated eye drops. The study showed sustained delivery of the HA for a prolonged period of time in both ex vivo and in vivo conditions, and may provide an alternative therapy for dry eye syndrome [163]. There is some evidence to suggest that HA may help in the acceleration of wound healing [164]. Perhaps the nanofibrous scaffolds developed from HA may serve to enhance the wound healing properties of HA for ocular therapy.

### 2.8. Chondroitin Sulfate

Chondroitin sulfate is one of the ECM components. The structure, molecular and nanomedicinal significance of the chondroitin sulfate as a biomaterial has been extensively reviewed by a number of experts [48,165]. Being a component of ECM, this biomaterial also plays an important role in cellular regeneration and wound healing. During wounds, chondroitin sulfate is also found to be upregulated in granulation tissue. An in vivo assessment was carried out to investigate the role of chondroitin sulfate in wound healing. With treatment of rabbit palatal fibroblast cells with chondroitin-6-sulfate, it helped in cellular growth adhesion in a dose-dependent manner. Whereas, the reverse effect was seen after treatment with a chondroitinase-mediated degradation of chondroitin sulfate. This study clearly indicated the importance of chondroitin sulfate in palatal wound healing [166]. Later, a chondroitin sulfate hydrogel was developed that was studied for its wound healing properties in rabbit maxillary mucosa. The histological examination of the wound showed that chondroitin sulfate was able to accelerate wound healing. The study concluded that chondroitin sulfate gels can serve as an important matrix or structural framework for fibroblast and epithelial regeneration [167].

Looking at the significance of chondroitin sulfate as biomaterial for cellular regeneration, it has been extensively explored for the development of nanofibrous scaffolds for improved wound healing. Jeannine et al. developed low-density 3D nanofibrous networks of PVA-methacrylate (MA)/PVA-chondroitin sulfate composites. The study showed that incorporation of chondroitin sulfate in fibers showed an enhanced synthesis of cartilage-specific collagen II synthesis in in vitro and in vivo conditions. These fibers were able to enhance cartilaginous tissue formation, suggesting its potential for use in articular cartilage repair [168]. Recently, PVA-chondroitin sulfate hybrid nanofibers loaded with combretastatin A-4 phosphate were developed as a drug delivery formulation. The study showed that the nanofibers were biocompatible with the mice fibroblast (L929) cell line [169]. These studies clearly indicate the use of chondroitin sulfates not only as drug delivery vehicles, but also for wound healing and tissue regenerative applications [170]. Moreover, it was recently suggested that chondroitin sulfate could also be used for ocular drug delivery. Abdullah et al. developed a chondroitin sulfate-CS hybrid nanoparticle system for the ocular delivery of bromfenac sodium. The study showed that the nanoparticle systems improved the corneal permeation and retention of the drug by 1.62 and 1.92 folds, respectively [171]. Perhaps chondroitin sulfate could also be useful for ocular drug delivery applications. The immunohistochemical analysis of rabbits after performing penetrating keratoplasty showed increased levels of chondroitin 6-sulfate at the repair site on day 7. They showed that large proteoglycans and chondroitin 6-sulfate may play important roles in corneal wound healing [172]. Moreover, there is some evidence that suggests that hybrid chondroitin sulfate scaffolds may aid in corneal wound healing [173]. Detailed analysis of chondroitin sulfate-porous nanofiber may help to understand its use in ocular healing.

### 2.9. Microbial Polysaccharides

Biopolymers are not restrained only to plant and animal sources. Many bioactive polymers of microbial origin have also been widely studied and employed in biomedical and nanoscience applications. Microorganisms are ubiquitous, and hence the polymers isolated from them are abundant and cost effective. Microbial polymers are derived from several species of fungi and bacteria. Pharmaceutically active sulfated polysaccharides have been a topic of pharmacological interest since a few decades ago. Sulfated polysaccharides extracted from various species of red algae, brown algae and green algae have been studied for innumerable biomedical activities, whereas microbial polymers have thus far been given the least importance in the field of medicine and biotechnology [48,174,175]. This is attributed to the antigenic nature of microbial polymers causing immune stimulation in human body. However, several microbial polymers have now been studied for their bioactive properties, and have been used extensively in industrial and biomedical applications.

Polyhydroxyalkanoates (PHA), known as microbial polyesters or bioplastics, are typical examples of polymers of bacterial origin. PHAs are produced intracellularly by certain bacteria, which may constitute 90% of their cell weight. PHAs share properties similar to those of synthetic polyolefins like polyethylene, polypropylene, etc., and fall under the class of thermoplastics of biological origin with high biodegradability and biocompatibility [176]. Polyhydroxybutyrate (PHB) is the most common form of PHA produced and used for industrial applications. Polyhydroxyvalerate (PHV), polyhydroxyhexanoate (PHH), and polyhydroxyoctanoates (PHO) are other examples of PHAs. Bacteria produce PHB as an energy storage molecule, and metabolize it during energy-deprived states. PHB is characterized by its high melting point, tensile strength, solubility in organic solvents, oxygen permeability, non-toxicity, etc. PHB crystals were added as a nucleating agent to increase the crystallinity of optically transparent plasticized poly (lactic acid)-bionano composite films, thereby enhancing its barrier properties [177]. PLA-PHB nanocomposite films reinforced with modified cellulose nanocrystals have been used for short-term food packaging [178]. Similarly, on functionalizing PHB crystals with different functional groups like aldehyde, amine etc., they can serve as a biocompatible carrier for drug delivery applications [179]. Just like many other natural polymers, PHB has also been studied for drug entrapment using various techniques, such as polymer precipitation and simple/multiple emulsion via solvent evaporation for the synthesis of micro and nanoparticles for drug delivery applications [180]. Apart from drug delivery, PHB-based scaffolds have been made using the electrospinning technique for tissue engineering and cell proliferation studies [181]. In a study by Gredes et al., PHB membranes were shown to be effective for bone regeneration through cell migration, proliferation, differentiation and vascularization [182].

Unlike plant and animal polymers, microbial polymers are not commonly used, for specific toxicological reasons. However, there are typical bacterial polysaccharides like mauran, gellan, etc. that are used in the biomedical industries. Mauran is a sulfated heteropolysaccharide extracted from *Halomonas maura*, which are moderately halophilic bacteria living under salt concentrations of 5–25%. Mauran is a combination of four different monosaccharides, glucose, galactose, mannose and glucuronic acid repeatedly bound via glycosidic linkages. They occur naturally in sulfated form with few phosphate groups attached to them [183,184]. Recently, mauran has been widely used for many bionanotechnology applications, including drug encapsulation and sustained delivery [185], nanofiber synthesis via electrospinning [186], stabilization of quantum dots [187], reduction and passivation of gold nanoparticles [188], etc. They have been well studied for their anticancer, antioxidant, antihemolytic, properties and have been found to be biocompatible with enhanced proliferation of mouse fibroblast cells during cell adhesion studies [189]. Functionalizing mauran with the fluorescent dye syproruby before nanoparticle synthesis with CS results in fluorescent nanoparticles that can be used for live bioimaging and cellular uptake studies [190]. In another study, mauran- and gellan-coated magnetic nanoparticles were analyzed for cancer therapy using drug targeting and hyperthermia [191]. Gellan gum is produced by a bacterium, *Pseudomonas elodea*. Chemically, gellan is an anionic heteropolysaccharide, with repeating units of a tetrasaccharide, composed of two units of d-glucose and one unit each of l-rhamnose, and d-glucuronic acid. Gellan has the ability to form strong clear gels at physiological ion concentration. Its good biological properties, along with its bioadhesive nature, have drawn enormous attention from nanotechnologists for several bionano applications, such as microparticles, nanoparticles, hydrogels, stabilizing agents, thickeners, emulsifiers, and coating agents [192]. Gellan gum beads in the form of micro- and nanoparticles have been widely used for drug delivery, and their effectiveness has been studied both in vitro and in vivo [193,194,195]. Similarly, the negative charge of the polymer facilitates the binding of heavy metals, and hence it has been successfully used as a sorbent [196]. Magnetic nanoparticles coated with gellan have also been used as an efficient sorbent that separates the heavy metals via magnetic separation and ion exchange [197]. Biocompatible gold nanoparticles can be synthesized using gellan as reducing/stabilizing agents. Cellular uptake and in vivo toxicity studies revealed that gellan-coated gold nanoparticles are devoid of cellular toxicity [198,199]. Large-scale aligned single-walled carbon nanotube-composed membranes were obtained from highly aqueous dispersions of gellan gum [200]. Furthermore, gellan gum-based hydrogels have received much attention in drug delivery and tissue engineering applications, as they mimic natural extracellular matrix to retain a large amount of water [201,202].

Bacterial cellulose is another kind of microbial biopolymer that has been receiving extensive attention over the last decade. The major difference between plant and bacterial cellulose is the absence of hemicellulose and lignin [203]. *Gluconacetobacter xylinus* is the most studied bacterium for cellulose production. Remarkably, microfibril formation and crystallization of bacterial cellulose can be altered through their culture conditions, which is an advantage over plant cellulose. Nevertheless, bacterial cellulose fibrils have equally good mechanical properties to those of plant-based cellulose materials. Cellulose fibrils of bacteria have excellent chemical stability and water-holding capacity due to their ultrafine fiber network. Bacterial cellulose on acetosulfation yields bacterial cellulose sulfate, and has been used to develop highly-transparent films with exceptionally good mechanical properties. Its application ranges from the biomedical and food industries to optoelectronics [204]. Moreover, applications of bacterial cellulose nanocrystals do not differ from those of cellulose nanocrystals with plant origin. Bacterial cellulose nanofibers incorporated with CdS quantum dots were used as an effective visible light responsive photocatalyst. The efficiency of CdS/bacterial cellulose hybrid nanofibers was proved by the photocatalysis of methyl orange dye after an exposure of 90 mins [205]. Silver nanoparticles incorporating bacterial cellulose membranes were used as antimicrobial wound dressings. Alternate dipping of bacterial cellulose membranes into silver nitrate or silver chloride solutions yields silver nanoparticle-bound antimicrobial films, which on antimicrobial assaying prove that they can inhibit the growth of *Escherichia coli* and *Staphylococcus aureus* [206]. The refractive index of bacterial cellulose (1.4–15) is similar to that of the human cornea (1.367). Hence, it is thought that it could be used as an excellent biomaterial for ophthalmic application. Recently, a BC-PVA composite was developed for use as potential corneal material. The study explains the chemical properties of the biomaterial for its use in ophthalmic applications. However, molecular or pharmacological significance is essential for the further development of the same [71].

## 3. Proteins

### 3.1. Collagen

Collagen is one the most abundant ECM animal proteins, and it serves as component of leather, glue, gelatin (discussed in the previous section) and others. The molecular structure (Figure 1) and other biochemical features of collagen as a biomaterial have been extensively reviewed elsewhere [207]. Collagen as a biomaterial has been extensively used for wound and ulcer dressings. Commercial products like partial purified skin (Gelfoam, Pfizer, New York, NY, USA), collagen sponge (Helistat, Integra Lifesciences, Plainsboro, NJ, USA; Instat, J&J, New Brunswick, NJ, USA), collagen fibers (Helitene, Integra Lifescience, Plainsboro, NJ, USA), collagen powder (Medifil, BioCore, Sydney, Australia), and collagen composite dressing (Fibracol, J&J, New Brunswick, NJ, USA) are some of the examples of collagen-based products used for wound healing purposes. The powdered form of collagen helps in promoting cellular recruitment, activation of the inflammation stage of wound healing, and helps in promoting new cellular growth. Another product, Biobrane^®^, which is a silicone membrane knitted with nylon membrane, both filled with porcine collagen, is useful for burn care.

Collagen skin can also serve as a substitute for the cryopreserved skin for the cure of diabetic wounds. Further, some studies suggest that collagen implants can also help in corneal wound healing. They show that corneal cells were able to have an excellent morphology when placed in a collagen matrix. Moreover, collagen-based systems modified with members of ECM, like fibrin and GAG, have been developed for the efficient growth of epidermal keratinocytes and fibroblast cells. Collagen mimetic peptides have also been developed for the development of hydrogels for various tissue engineering applications [207]. At a cellular level, most collagen products reduce the elastase level in the wound microenvironment and disrupt the cycle of chronicity. Native collagen helps in the promotion of angiogenesis and increases fibroblast chemotaxis, which plays a significant role for wound healing purposes. Moreover, certain studies show that fibroblasts work efficiently when anchored to the 3D architectural structure of collagen [208,209]. Collagen as a biomaterial has also been extensively studied for its biogredability by metalloproteinases, such as MMP-1, MMP-2, MMP-8, MMP-13 and MMP14. The high biocompatibility and biodegradability of collagen makes it an ideal biomaterial for application in a variety of biomedical applications [207]. Development of nanofibrous scaffolds of collagen has been extensively reviewed [210,211]. Collagen has been used in both its native and composite state with PCL, PLA, PEO, P(LLA-CL), PHBV for the development of nanofibers through an electrospinning process [211]. A study of collagen type-1 nanofibers was undertaken, and showed them to have accelerated wound healing properties. The results showed that nanofibrous scaffolds of collagen could be ideal candidates for wound healing applications [212]. Recently, electrospun tilapa collagen was developed for wound healing. The study showed that nanofibers promote human keratinocyte (HaCaTs) proliferation and stimulates epidermal differentiation by upregulation of regulation gene expression for involucrin, filaggrin and type 1 transglutaminase. Under in vivo conditions, these fibers were also able to facilitate rat skin generation [213].

Collagen is also one of the most abundant proteins in the human cornea. In 1987, a preliminary study showing the effect of a collagen bandage lens on corneal wound healing was published. It was shown that after 24 h of treatment, rabbit eyes with collagen bandages showed improved corneal wound healing compared to the control group. The treated eyes were found to have reduced stromal edema at the wound site [214]. Later, collagen-based hydrogels were developed, which were found to be biodegrable and biocompatible with proliferation and differentiation of limbal epithelial cells. Moreover, some clinical studies also suggested that corneal re-epithelialization could be achieved with treated with collagen hydrogel [67]. These studies clearly indicate the importance of collagen as a biomaterial in corneal and ocular delivery. Many collagen-derived products have entered into the market. Perhaps a similar fate can be expected for other collagen nanoscaffold-based products in the field of wound dressing and corneal regeneration.

### 3.2. Gelatin

Gelatin is a typical example of a polymer protein derived from collagen through partial hydrolysis. Collagen (discussed above) is extracted from animal by-products especially from the connective tissues of domesticated animals like cattle, pigs, etc. During the hydrolysis of collagen, natural inter- and intramolecular covalent bonds are broken, leaving individual strands to give a simpler form called tropocollagen. Further denaturation of tropocollagen by breaking the hydrogen and hydrophobic bonds gives a heterogeneous proteinaceous material, gelatin [215]. There are several methods of collagen hydrolysis, including acid, alkali and enzyme treatment, as well as thermal degradation. Among these, acid and alkali treatment are mainly applied in the commercial production of gelatin. There are two major types of gelatin available commercially: gelatin type-A, and gelatin type-B; however, the molecular heterogeneity of gelatin is a big issue for preparing a highly homogenous polymer formulation. The presence of both an amino and carboxylic group makes it an amphoteric compound, and it is readily soluble in hot water. The viscosity is greatly affected by its type, concentration and temperature. Gelatin is widely used in the food and pharmaceutical industries due to its gelation property, and has been widely employed for several bionano applications. A variety of polymers can be blended with gelatin for different applications, although the pH and ionic strength of the mixture plays a key role. Here, we discuss the major role of gelatin as a biocompatible polymer for bionanoscience applications [216].

#### 3.2.1. Nanotechnology Applications of Gelatin and its Composites

Gelatin as a natural biopolymer has gained a lot of interest in the biomedical industry due to its biocompatibility, biodegradability and less production cost. It has the ability to bind various target moieties due to the availability of too many active sites. With respect to these advantages, gelatin is widely used for the preparation of microspheres and nanoparticles for drug and gene delivery. Based on the chemistry of the combination polymer with gelatin, several techniques have been employed for gelatin nanoparticle preparation, including emulsification, solvent extraction, coacervation-phase separation, desolvation, nanoprecipitation, layer-by-layer coating, reverse-phase micro emulsion and self-assembly [217]. Han et al. demonstrated the synthesis of gelatin-based amphiphilic copolymer nanoparticles with a hydrophobic and hydrophilic moiety in a single nanocomposite. Gelatin was used as the hydrophilic segment and poly (lactide) and 1,2-dipalmitoyl-*sn*-glycero-3-phosphoethanolamine (DPPE) (gelatin-*co*-PLA-DPPE) as the hydrophobic one for encapsulating an anticancer test drug doxorubicin via a double emulsion/ nanoprecipitation method [218]. In a similar study, a core-shell nanoparticle based on gelatin, iron oxide and calcium phosphate was successfully fabricated for anticancer drug delivery and magnetic resonance imaging. Doxorubicin was encapsulated via electrolytic co-deposition of calcium phosphate to form a shell over gelatin-iron oxide core. These gelatin-based core-shell nanoparticles were developed as a promising multifunctional nanodepot for therapeutics and diagnostics [219]. Electrostatic interaction between negatively charged gelatin as well as positively charged molecules and vice versa is always encouraging for making ideal nanocomposites [220]. A positively charged EGFR2R-lytic hybrid peptide with excellent antitumor and cytotoxic activities is bound to gelatin electrostatically to form hydrogel nanoparticles. This nanoparticle composite showed higher antitumor activity and greater circulation time during release kinetic studies. This has been attributed to the slow and controlled release of peptide from the gelatin hydrogel beads [221].

Gelatin is also used for nucleic acid delivery. A variety of gelatin-based nanocarriers and microspheres have been developed for controlled and sustained delivery of drugs, peptides and proteins; apart from that, they have been well studied for siRNA delivery and gel-based tissue engineering applications. Various nanocarriers have been considered for siRNA delivery; however, strong cationic polymer-based carriers were found to be most appropriate for effective siRNA delivery [222,223]. Glutaraldehyde cross-linked gelatin nanospheres were used to encapsulate siRNA and fluorescently labeled to study the internalization and gene silencing process in vitro. It was observed that the suppression of gene expression was in accordance with the slow degradation of gelatin nanospheres and the consequent release of siRNA [224]. Gelatin nanoparticles were used for tumor-targeted delivery of polymerized siRNA in tumor-bearing mice. Thiolated gelatin was used to encapsulate the polymerized siRNA without losing its gene silencing property and prevented from enzymatic degradation. This biocompatible nanoformulation efficiently delivered siRNA to red fluorescent protein expressing melanoma cells, and down-regulated the target gene expression successfully [225].

Modifying gelatin with chemically compatible polymers is always promising for siRNA delivery and gene knockdown. Gelatin-Polyethyleneimine (PEI)-based core-shell type nanogels were made for siRNA delivery into HeLa cells, to study argininosuccinate synthetase gene knockdown. Gelatin-PEI nanogel was compared with a commercially available transfection agent, Lipofectamine, and was found to be highly efficient in its gene-silencing ability and uptake efficiency [222]. Apart from the delivery of siRNA, plasmid DNA transfection was also achieved by culturing mesenchymal stem cells in the gelatin/β-tricalcium phosphate three-dimensional scaffolds containing luciferase plasmid DNA complexed with spermine-pullunan [226], thus showing the versatility of gelatin hydrogels and scaffolds in bionanoscience applications.

#### 3.2.2. Gelatin Nanoconstructs in Tissue Engineering and Regeneration

The colloidal gel made using oppositely charged gelatin nanoparticles were entrapped with two different growth factors to demonstrate dual or multiple deliveries of proteins or peptide in vivo. An angiogenic basic fibroblast growth factor and osteogenic bone morphogenetic protein-2 were delivered by means of injectable colloidal gelatin gels and tested for bone regeneration [227]. The results from Wang et al. also support the multiple encapsulations of multiple proteins or growth factors for regenerative medicine applications [228]. Injectable hydrogels are always fascinating in their applications, especially for tissue regeneration and engineering. Colloidal gel composites incorporated with different organic and inorganic nanoparticles are an attractive class of hydrogels for injecting, shape-specific fabrication, and bulk scaffold synthesis [229]. Unlike synthetic polymer gels, gelatin-based hydrogels are highly encouraging for the growth and differentiation of mammalian cells [230]. Nevertheless, several synthetic polymers are conjugated with gelatin for cell morphogenesis and migration studies. Micropatterned gelatin methacrylate hydrogels were used for studying three-dimensional endothelial cord formation with versatile geometric features. As a preceding step to tubulogenesis, endothelial cord formation was studied with varying shapes, and these gelatin microconstructs were found to be appropriate for engineering tissue constructs [231,232]. Gelatin-based micro and nanofiber scaffolds have been widely used for culturing cell and tissue grafts. Photo- cross-linked gelatin membranes incorporated with growth factors like neurotrophins, BDNF, NT-3, etc. and extra-cellular membrane proteins were demonstrated to be efficient for the adhesion, alignment and proliferation of schwann cells, and found highly promising for spinal cord injury repair [233]. Furthermore, Gelatin/nanoceria nanocomposite fibers were developed as antioxidant scaffolds for neuronal generation [234]. PEC of gelatin-CS was also explored for the development of nanobioglass 3D scaffold for bone tissue engineering [235]. This indicates the importance of gelatin-based nanoscaffolds for cellular regeneration and growth.

**Figure 8:**
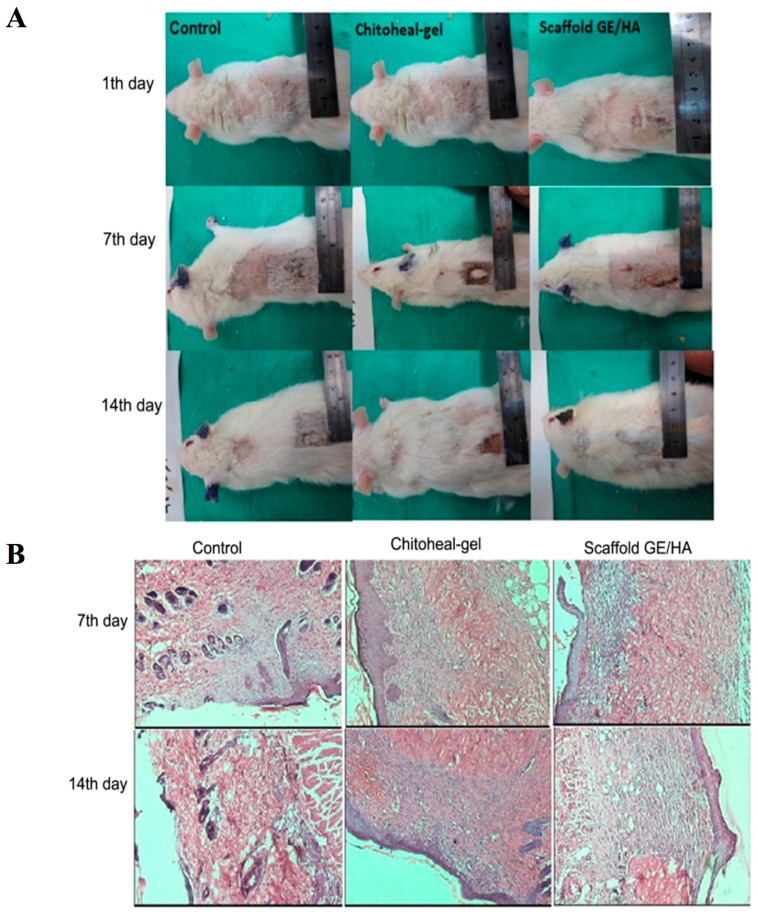
In vivo Evaluation of Gelatin/Hyaluronic Acid Nanofiber as Burn-wound Healing and Its Comparison with ChitoHeal Gel. (**A**) It can be seen that at the end of 14th day GE/HA nanofibrous scaffold is able to completely heal the wound in comparison to chitoheal gel control. (**B**) The H&E stained section of granulation tissue of control, chitoheal gel and GE/HA nanofibrous scaffold shows that irregular collagen bands that are evident in control are absent in GE/HA nanofibrous scaffold. Reproduced with permission from [240].

Gelatin as a bulk material shows impressive wound healing properties. During the wound healing process, the Epidermal Growth Factor (EGF) plays a critical role in stimulation, proliferation and migration of keratinocytes. EGF not only helps in the formation of granulation tissue, but also stimulates fibroblast motility, which induces cell-cell adhesive properties consistent with the epithelial phenotype, thereby enhancing wound closure. Gelatin avoids proteolysis of EGF by proteases. Moreover, a recent study has also showed that Gelatin can act as biodegradable carrier for the sustained delivery of EGF [236]. This shows dual-mode cellular regeneration, owing to the biological characteristics of gelatin. Gelatin has been extensively employed for controlled delivery of anti-infective and anticancer drugs under a variety of nano designs and combinations. Gelatin has been considered an excellent carrier for the delivery of cells for tissue engineering and wound healing applications [237]. Chong et al. developed a novel PCL-gelatin nanofibrous scaffold called Tegaderm™ [238]. In vitro evaluation of the scaffold showed cellular adhesion and growth. They evaluated their gelatin-based nanofibrous scaffold as wound dressing material on a Porcine wound healing model. The study showed that all test groups treated with Tegaderm were able to get sufficient recovery and healing. They did not show any signs of infection or adverse reaction. The Tegaderm group showed complete healing on the 28th day of treatment [239]. Recently, in vivo evaluation of gelatin/hyaluronic acid hybrid nanofibers was also evaluated for wound healing purposes in comparison to Chitoheal gel. At the end of the 14th day, it was shown that the nanofibrous scaffold provided improved the wound healing property compared to the gel-based formulation (as shown in Figure 8) [240].

Gelatin as a biomaterial has also been commercially explored for its potential for ocular therapy. Gelatin film called as GELFILM^®^ is an ophthalmic film with an approximate thickness of 0.075 mm that is used as an absorbable implant in ocular surgery such as the glaucoma filtration operation. The material has been shown to provide considerable biocompatibility. Recently, a gelatin hydrogel was developed for delivery of EGF for corneal wound healing. The study showed complete wound closure after 96 h of treatment with EGF-CGH (cationized gelatin hydrogel) film [241]. In addition, Gelatin-PVA blend is also used for the delivery of antibiotics to the eye. This study clearly shows the significance of gelatin material in corneal wound healing [242]. Gelatin is also blended with various synthetic polymers (as discussed in Table 4) to create nanofibers that have been proven to be potential nanoformulations for ocular wound healing. These studies clearly show that nanofibers play a critical role in wound healing.

### 3.3. Casein

Casein is a potential milk protein (19–25 kDa) that is used for drug delivery applications in the form of micelles of size 100–200 nm. Four different casein phosphoproteins have been identified, αS1, αS2, β and k-casein, respectively. There are distinct hydrophobic and hydrophilic domains in the casein protein structure. Based on several functional groups (phosphate, carboxyl and amino groups) present, they can be tailored to bind various other polymers of opposite charge to fabricate micro and nanoparticles. Casein microparticles, nanoparticles, hydrogels, micelles, etc. are widely employed for various bionanoscience applications like drug delivery, tissue engineering and stabilization of metal nanoparticles [243]. Phosphopeptides obtained from digestion casein (casein phosphopeptide, CPP) have been shown to enhance the absorption of calcium (Ca). It was shown that these phosphopeptides prevented the precipitation of Ca phosphate salts, and it has been suggested that it can maintain the high concentration of Ca in intestinal lumen. These studies clearly suggest the importance of casein protein in the growth and development of bone tissue. Perhaps the use of this protein in the form of nanocomposite can also be used for tissue repair/ regeneration or wound healing [244].

#### 3.3.1. Nanotechnology Applications of Casein and Its Composites

Casein serves as a natural known vehicle that delivers amino acids and calcium ions from mother to baby through milk. Lack of a three-dimensional rigid structure causes changes in geometry and size when there is a change in temperature, pH, water activity, hydrostatic pressure and ionic strength [243]. As stated previously, based on the availability of several functional groups (phosphate, carboxyl and amino groups) present, they can be tailored to bind various other polymers of opposite charge to fabricate micro and nanoparticles. Casein microparticles, nanoparticles, hydrogels, micelles, etc. are widely employed for various bionanoscience applications like drug delivery, tissue engineering and stabilization of metal nanoparticles [245]. Cross-linked casein nanoparticles were used for the prolonged and controlled delivery of poor soluble anticancer drugs like flutamide. After crosslinking with a polyanionic sodium tripolyphosphate, casein nanoparticles were used for drug delivery applications, and the in vivo pharmacokinetics were studied [246,247]. The report says that PLGA-casein hybrid nanocarriers can entrap dual drugs with distinct hydrophobic and hydrophilic natures and sequentially deliver under in vivo conditions. Polymer protein nanoparticles were prepared using a simple emulsion-precipitation method [248]. Bioimaging based on nanoparticles is another important application in the field of nanotechnology. Bare iron oxide nanoparticles are demonstrated to be excellent Magnetic Resonance Imaging (MRI) contrast agents. However, its toxicity hinders its direct application in the biomedical industry. Just like several other biopolymers, casein was also studied for the stabilization and coating of iron oxide nanoparticles for MRI high contrast enhancement, as well as for efficient cellular targeting. As a result of casein’s functionalization over magnetic nanoparticles, highly efficient MRI contrast agents with excellent water solubility, colloidal stability, and biocompatibility were obtained [249]. Casein, as a versatile protein polymer, has been thoroughly studied for a variety of bionano applications due to its high affinity for binding ions and small molecules, as well as itsnatural ability to self-assemble as micelles.

#### 3.3.2. Casein Nanoconstruct in Tissue Engineering and Regeneration

Casein has been prominently explored for use in the oral delivery of drugs. However, its use in the form of nanoconstruct for tissue engineering is relatively new; for example, electrospinning of casein in the form of nano composite fibers [250]. This provides an interesting opportunity for scientific researchers to develop nano porous and fibrous scaffolds for bone tissue regeneration purposes. CPPs can be used as nucleation sites for binding calcium ion promoting hydroxyapatite (HA) formation for bone tissue engineering. Hence, a carboxylated graphene oxide (CGO)-CPP nanocomposite was developed that can serve as a scaffold for HA coating on its surface and can, in turn, help tissue engineering applications [251]. Casein has also been explored for the formation of gel along with albumin to develop a porous 3D scaffold for tissue engineering purposes [252]. Casein protein plays an important role in cellular homeostasis by maintaining balance in growth and apoptosis. α-isoform of casein protein exhibits a tumor suppressor function by activation of the STAT1 signaling pathway and helps in preventing breast cancer [253]. On the other hand, glutaraldehyde, the cross-linked casein (GCC) conduit, helps in nerve repair activity [254]. Unlike certain polysaccharide biomaterials, casein does not possess any anti-infective properties. Perhaps PEC of casein can be explored to identify its anti-infective potential based wound healing capability.

### 3.4. Silk Fibroin

Silk worms synthesize a fibrous composite protein called silk, composed of two major components: (a) fibroin, and (b) sericin. Of the two silk proteins, fibroin forms the major structural component of fibrous silk, whereas sericin covers the fibroin to form a composite silk protein fiber. Fibroin has been relatively more explored for its biomedical applications. Silk fibers are mostly obtained from silkworms (*Bombyx mori*) and have a diameter of around 10 to 25 μm. Apart from *B. mori*, there are eleven other sources for obtaining silk, with variations in their mechanical and chemical properties [255]. The core structure of fibroin contains three major components, a heavy chain (390 kDa), a light chain (26 kDa) and a glycoprotein (25 kDa) unit. It exists as a fibroin protein complex with around 5263 amino acid residues and a highly conserved hydrophilic N-terminal, a hydrophilic C-terminal domain and 12 linker regions [256]. The full-length molecular structure of various domains of silk fiber from *B. mori* remains unknown.

#### Silk Fibroin (SF) Nanoconstruct in Tissue Engineering and Regeneration

Like other biomaterials, silk is a biodegradable soft material that has been explored extensively for various biomedical applications. Silk scaffolds for tissue engineering and repair have been studied extensively for the degradation pathways under both in vitro and in vivo conditions. Multiple studies suggest that enzymes like Protease XIV, XXI, actinase, trypsin, and chymotrypsin/collagenase IA are responsible for the degradation of scaffolds produced from silk [255]. In vivo degradation of 3D scaffolds of silk in Lewis rats showed that they disintegrate within a few weeks and completely disappear 1 year after implant [257]. Studies have also suggested that osteoblast and osteoclast cells can degrade silk scaffolds via expression of metalloproteinases (MMPs) and integrin [258]. All these studies clearly suggest that silk-based scaffolds exhibit extreme ease of biodegradability and in vivo clearance without significant immunogenic reactions. This makes this molecule an excellent candidate for wound healing or tissue repair applications.

**Figure 9:**
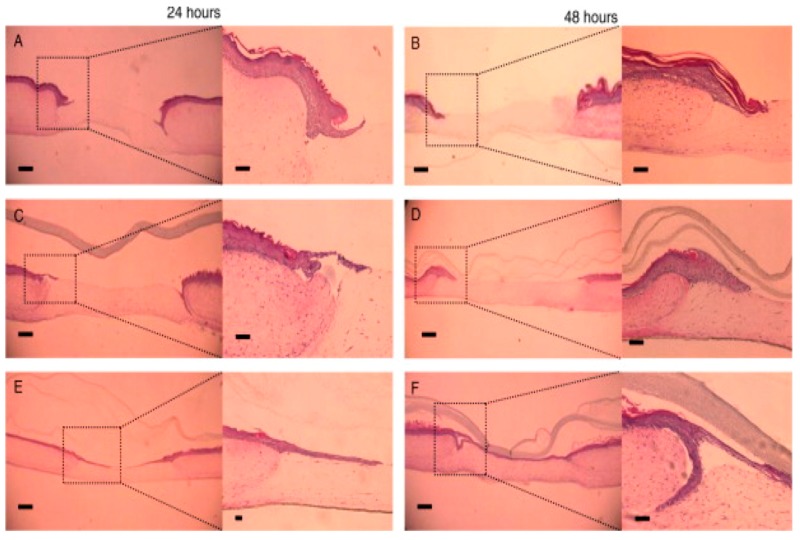
Hematoxylin and Enosin staining of wound after 24 and 48 h of treatment with silk nanofibers. (**A**,**B**) the control without silk dressing on the top of the wound, (**C**,**D**) samples of silk mat without EGF, (**E**,**F**) samples of silk mat consisting of EGF. It can be seen that silk mat with EGF shows complete closure of the wound after 48 h of incubation. Reproduced with permission from [261].

It is essential to understand the mechanism of wound healing governed by silk proteins. Celia et al. studied the cellular mechanisms by which silk proteins may govern wound healing. They used model human cell lines to show wound closure using a standard scratch assay. Their studies indicated that at a molecular level, MEK (mitogen activated protein kinase), JNK (c-Jun N-terminal Kinases) and PI3K (Phosphatidylinositol-4,5-bisphosphate 3-kinase) pathways might be involved in fibroin- and sericin-mediated cell migration and wound closure [259]. Perhaps a similar mechanism is employed by nanoscaffolds developed using silk proteins (fibroin and sericin). Recently, SF was also studied for the cell migration, proliferation and adhesion of corneal cells. It was observed that SF increased migration rate by 50% and produced an approximately 60% increase in cell proliferation of human corneal limbal-epithelial (hCLE). This indicates that SF as a protein can also be useful for corneal wound healing applications [260]. Apart from the use of native silk protein as a wound healer, at a molecular level, they are extensively used as a nanofibrous carrier for various growth-promoting molecules, like epidermal growth factor (EGF). The study showed release of EGF over a period of 6 days. It showed that EGF-conjugated SF (blended with PEO) nanofibers provided a 3.5-fold increase in the wound closure rate compared to native fibroin by the end of 48 h (as shown in Figure 9) [261].

It is also shown that type 1 collagen also plays an important role in wound healing by promoting cell attachment, proliferation and differentiated function in tissue culture. Type 1 collagen is a type of extra-cellular matrix protein (ECM) with arginine-glycine-aspartic acid (RGD) residues that are recognized by integrins. Integrin is a cell surface receptor protein that is expressed by a variety of cell types, and helps in cell-cell and cell-ECM adhesion. Collagen type 1 may help in integrin-mediated cell-cell adhesion, migration and proliferation [212]. In order to assess this effect, ECM protein (including collagen type 1)-coated SF nanofibers were developed. To achieve cellular attachment and growth, normal human keratinocytes and fibroblasts were used. The study clearly indicated that collagen type 1-coated SF nanofibrous composites were able to promote cell growth and adhesion [262]. A similar collagen-SF nanofibrous scaffold was also found to be useful in the growth and development of hepatocyte (HepG2) cells. This nanofibrous template of SF-collagen may serve as an interesting candidate for wound healing purposes. SF has also been used in nanocomposite states with polysaccharides (AGs and CS) for skin, cartilage, tendon and ligament tissue engineering purposes.

The SF protein structure plays a crucial role in cell adhesion and proliferation. Apart from the importance of c-terminus domain on cell adhesion [263]. Recently, structural insights into the importance of N-terminal domain of SF (FibNT) have been provided (shown in Figure 1). It has been suggested that pH and salt concentration play an important role in the formation of globular structures that can lead to the formation of silk fibers under in vivo conditions. It has also been shown using DLS and SEM that at pH 5 it is possible to attain globular nanoparticles with N terminal domain of fibroin with particle sizes of around <100 nm [256]. This indicates that FibNT may serve as an interesting biomaterial for nanomedicine along with full-length SF.

Moreover, SF-based transparent films of thickness 30 ± 9.7 μm were developed. These films showed considerable biocompatibility towards keratocytes and promoted growth of corneal cells. The study also demonstrated the acceptability of films under in vivo condition [77]. Further, aloe vera-blended SF transparent scaffolds were also created for corneal wound healing applications. The study concluded that these transparent scaffolds supported growth of corneal epithelial cells (CECs) by maintaining the morphology and normal function of the same. They also demonstrated the successful implantation of scaffolds to corneal stroma without any major inflammatory response, and they may serve an important role for patients suffering from corneal diseases [264]. Such SF micro scaffold thin films have been extensively studied and reviewed as biomaterials for the corneal wound healing purposes. However, the study of nanoconstructs for ocular therapy has been relatively less explored [265].

### 3.5. Keratin

**Figure 10:**
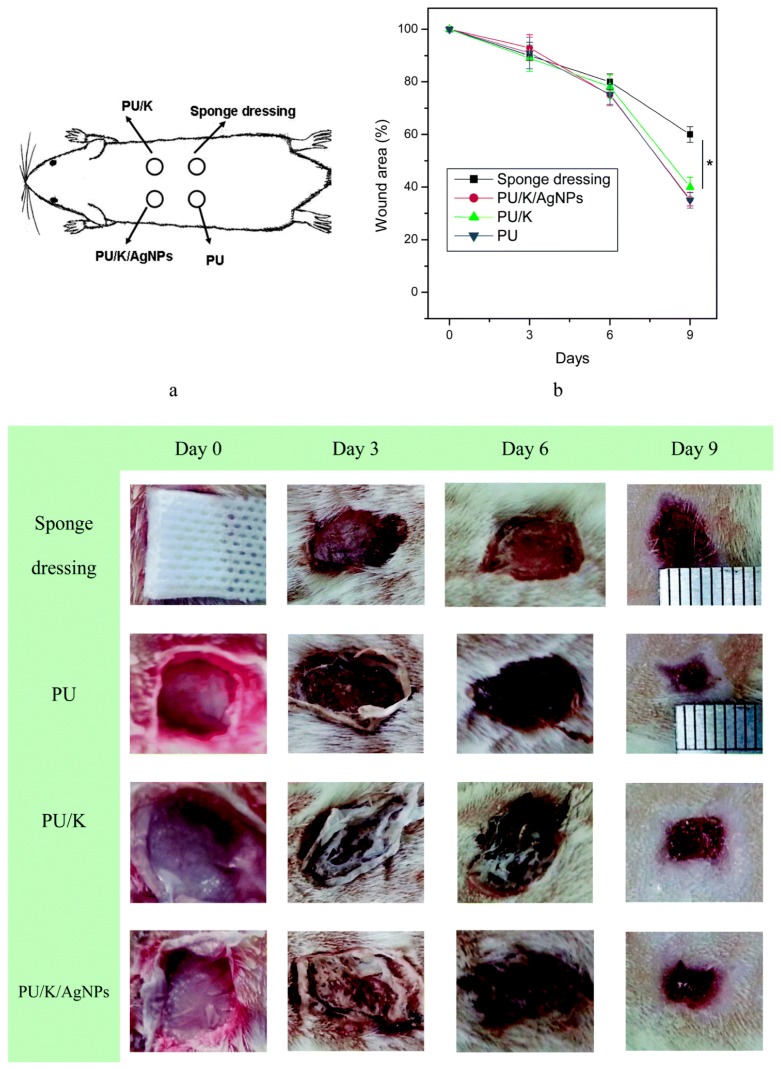
(**a**) Shows representative figure that depicts wound treatment on mice model (**b**). It shows that reduction of wound area for mice treated with test and control products over period of 9 days. It clearly shows closure with polyurethane keratin nanofibers (PU/K) with and without AgNPs in comparison to standard sponge dressing. It can be seen that both PU/K and PU/K/AgNs dressing shows wound healing on 9th day of treatment. The area of wound was reduced to 30% in case of PU/K- and PU/K/AgNP-treated rats. Reproduced with permission from [267].

The epidermis’ performance of its most fundamental function as a protective barrier is undertaken with help provided by the integrity of keratin networks. Keratin is a cytoskeletal protein (insoluble in water) with a molecular weight in the range of 44 to 66 kDa. There are around 108 different subtypes of keratins that are classified in two groups as type I (acidic) and type II (alkaline). These types can be further classified, depending upon their site of expression, as epithelial keratins, hair follicle keratin-specific epithelial keratins, and hair keratins [266]. Keratins are generally expressed in pairs of type I and II conformers to form heterodimers that form the keratin filaments or tetrameric bundles (shown in Figure 10).

#### Keratin in Tissue Engineering and Regeneration

Epithelial cells produce different types of keratins under various stages of growth. For instance, cells in the basal compartment that have the capacity to proliferate, such as stem cells, produce keratin subtypes K5 and K14. Post mitotic epithelial cells express K1 and K10. There is a strict balance of proliferation, differentiation and desquamation maintained in epidermis. However, during injury, epithelial cells have the capacity to escape the differentiation pathway and take over the migration, enhancing proliferation to produce the necessary epithelium cover. Under such conditions, a new set of keratins, such as K6 (type II), K16 (type I) and K17 (type I), are expressed. To be specific, a study suggested that 6 h after injury to the human epidermis there is a strong induction of K6 and K16 (type II and I) subtypes and their re-localization in sub compartments at the start of cellular re-epithelialization [268]. Hence, K6 and K16 may play a crucial role in chronic wound healing mechanisms [269]. In addition, a study on mice models confirmed that knockdown of MK6 keratin gene showed a delay in re-epithelization of hair follicles after superficial wound formation [270]. The study showed that the absence of MK6 prevented the migration and proliferation of follicular keratinocytes under in vitro conditions. Keratin subtypes like K6, K16 and K17 are expressed by various cells such as hair follicles, palms, soles, nail beds, mucous epithelia such as the oral cavity, esophagus, trachea, and vaginal and anal epithelia. There is evidence that shows that the bundles of epithelial K8 and K18 may also play a crucial role during the healing process [271]. Also, a study suggested that K6/K16 and K3/K12 could also play an important role in the corneal epithelial regeneration [272].

Given the importance of keratin in wound healing and tissue regeneration, it is logical that keratin be used as a natural biomaterial for the development of scaffolds for wound healing or tissue regeneration applications. In a study, the hydrogel-based formulation of keratin extracted from wool and human hair was prepared. The study showed that a human hair-extracted keratin-based dressing provided relatively better wound healing property in male Sprague dawley rats (8 weeks old) at the end of 14 days compared to wool-extracted keratins. Use of keratins in the development of nanofibrous scaffolds is relatively new. As discussed previously, fibroin nanofibrous scaffold helped in the growth and development of keratinocytes (epidermal cells) that produce keratin and fibroblasts. Hence, looking at the significance of fibroin in cellular adhesion and proliferation, Marina et al. developed SF-keratin composite nanofibers. The study only provided characterization of composite nanofibers and film [273]. Keratin is commonly used in a composite state with synthetic polymers. Keratin-PCL composite nanofiber membrane was developed. It was assayed to check the cellular adhesion and spreading characteristics. The study showed that 3T3 fibroblast cells grew well on the surface of the keratin-PCL nanofibers after 24 h of incubation. The study also showed that increased relative concentration of keratin from 0 to 30 parts slightly enhanced the cell viability [274,275]. Similarly, keratin-PEO blend nanofibers were reported in the International Conference on Nanotechnology and Biosensors conference proceedings. The study showed that the fibers were able to provide enhanced NIH 3T3 cell adhesion and proliferation [276]. Along similar lines, Wang et al. developed polyurethane/keratin/AgNO_3_ (silver nitrate) bio composite nanofibrous mats for antimicrobial-based wound dressing to enhance cellular adherence and proliferation. The composite mat was able to provide an antimicrobial effect against *E. coli* (1.9 mm) and *S. aureus* (3.1 mm) zone of inhibition assay. The in vivo wound healing test on female Sprague-Dawley rats showed complete wound closure on the 9th day of incubation with the nanofibrous mat which was better than the result obtained with the sponge dressing. These studies clearly indicate the importance of keratin as a biomaterial and as an essential element for wound healing purposes (shown in Figure 10) [267].

### 3.6. Laminins

Laminins are the large family of multi-domain trimeric basement membrane glycoproteins that contribute to the complex structure of ECM. It helps in cellular adhesion, differentiation, migration, phenotype stability and resistance to anoikis [277]. Laminins are important for early embryonic development, organogenesis and have important functions in several tissues including muscle, nerve, skin, kidney, lung and the vasculature. In wound healing, laminins play an important role in re-epithelialization and angiogenesis. For re-epithelialization, laminins play the specific role of providing the necessary substrate for epithelial keratinocytes to cover the wound and establish an intact epithelial barrier. For skin and cornea, the major laminin component is α3Aβ3γ2 (LM3A32 or LM33), with lesser amounts of LM511, LM3A11, and LM3B32, which play an important role in the healing process by affecting the motile behavior of keratinocytes. Furthermore, the endothelial basement membrane of smaller vessels consists of the LM411, whereas dermal vessels also consist of LM511 and LM3B11. Hence, they play a major role in blood vessel growth and maturation, and help in angiogenesis [278].

The fibrous network of laminin alone can retain the conformational state of the basement membrane, and is sufficient for promoting cell adhesion and growth. As a result, Rebehah et al. developed a laminin nanofiber mesh that can mimic the bioactivity of the basement membrane. Unlike other nanofibers, these laminin nanofibers (LNFs) do not require chemical fixation and can be used in their native state. The geometry of the LNFs was retained for 2 days, and cell culture studies showed growth of PC12 cells with extended neurites without nerve growth factor stimulation. Also, these fibers were able to enhance the rate and quantity of human adipose tissue stem cells (ASCs). ASCs were viable on the mesh for 3 days in serum free media on LNFs [279]. Further, Laminin-PVA hybrid nanofibers were developed for regenerative medicine. It was found that human embryonic stem cells remained under undifferentiated condition when cultured with LNFs. Similarly, the study also confirmed that mouse embryonic stem cells were able to maintain their colony for twice as long without passage compared to the negative control (PCL fibers) [280].

It was found that human corneal endothelial cells (HCECs) express LM5 receptor α3β1 integrin. It was shown that the LM5 sub type of laminins promotes adhesion, migration and moderate proliferation of cultured HCECs. This may be an important cue for the use of laminins in ocular wound healing applications [281]. Looking at the importance of laminin in corneal wound healing, Uzunalli et al. developed bioactive peptide nanofibers, made by self-assembling peptide amphiphile molecules containing laminins. The study clearly showed that human corneal keratocyte cells cultured on the nanofibers were able to retain their characteristic morphology. Moreover, this study also showed the use of the nanofiber system for the repair of damaged rabbit corneas. These results clearly indicate that laminin-mimetic peptide nanofibers can provide a promising injectable, synthetic scaffold system for corneal stroma regeneration [282], which in turn indicates the importance of laminin for a variety of wound healing purposes.

### 3.7. Fibronectin

Fibronectin is a macromolecular glycoprotein (Figure 1) that works as an adhesive molecule in the wound healing process, and also particularly helps in the formation of ECM. Use of this biomaterial in nano drug delivery is relatively new [283]. It is found in all tissues and it is important for carrying out various cell matrix interactions. The detailed role of fibronectin in the wound healing process has been extensively reviewed elsewhere [284,285]. The role of fibronectin in corneal wound healing was established in 1981, where Fujikawa et al. suggested that fibronectin and fibrin play important roles in epithelial cell migration and temporary adhesion to the surface during corneal wound healing [286]. The fibronectin that appears at the site of corneal injury provides the necessary matrix to support the migration of remaining epithelial cells so that they can cover the defect [287]. Further, when topical application of fibronectin through eye drops was applied to rabbits whose cornea was damaged with iodine vapor, treatment with fibronectin provided a healing rate of 1.62 ± 0.33 sq mm/h, whereas the control eyes had healing rate of 1.30 ± 0.35 sq mm/h [288]. This clearly indicates the importance of fibronectin as a biomaterial in the repair of skin and ocular wounds.

Hence, with regard to the natural healing capacity of fibronectin, it has been used for the development of nanofibrous scaffolds that could aid in improving the healing process. Emille et al. developed a fibronectin mimetic peptide sequence that consists of RGD domain. These nanofibers were extensively studied for their chemical properties [289]. However, their pharmacological evaluation remains unexplored. The study showed that the RGD domain containing laminin nanofibers showed relatively better growth for human corneal keratocyte cells compared to fibronectin nanofibers. Perhaps the strategy of developing peptide mimetic nanofibers is only useful for tissue wounds other than ocular ones. Further, PLGA/collagen nanofibers loaded with recombinant fibronectin (FN)/CDHs was developed for bone tissue engineering. The study showed that human bone marrow mesenchymal stem cells (hMSCs) were able to adhere and differentiate in the presence of nanofibers. They showed that FN/CDHs can induce the differentiation into osteoblast cells [290]. Apart from being a part of the core shell structure as discussed previously, fibronectin was explored for its tissue engineering purpose by attaching itself on the surface of the PLGA nanofibers. The study showed that human periodontal ligament (hPDL) cells exhibited improved adhesion and were able to attain homogeneous colonization when cultured with surface-attached fibronectin PLGA nanofibers [291]. These studies suggest that nanofibrous constructs from fibronectin biomaterial may be used for a variety of cellular regeneration and perhaps wound healing applications.

## 4. Conclusions

The use of bionano science in the development of the pharmaceutical and biotechnology industry has been increasing exponentially. There are around 40 nanomedicines (belonging to categories like liposomes, lipid based formulations, PEGylated formulation, nano crystals, polymeric nanoparticles, protein nanoparticles, surfactant-based formulations, metal-based nanoparticles, virosomes) that have been approved by FDA in last few decades [292]. This indicates the significance of the soft biomaterial in biomedical application. Moreover, some sources have also predicted an increase in the market of biomaterials to USD 149.17 billion by 2021, from 70.90 billion in 2016. This is because of the use of these soft materials in nanomedicine, orthopedics, cardiovascular applications, wound healing and dentistry [293]. It can be observed that both polysaccharides and protein-based biomaterials have been extensively explored for the biomedical applications. It can be seen that blends of the polysaccharide-protein/protein-synthetic polymer/polysaccharide-synthetic polymer have been explored for improving the physicochemical and biological properties of nano or micro scaffolds for biomedical applications. In the field of regenerative medicine, the FDA has approved only ten products belonging to three categories—biologics, cell-based medical devices and biopharmaceuticals—for use for regenerative applications. Normally, drug development or introduction of pharmaceutically active ingredients to market takes dozens of years, with an average cost of around $802 million to $2.6 billion per molecule. However, the introduction of medical devices (a broad category) includes non-cellular products reach the market with 3–7 years of development [294]. This makes nano healing materials interesting products for quicker introduction onto the market. In the current review, we have tried to explain the importance of various natural (carbohydrate and protein) biomaterials, and their importance for nanotechnology and regenerative medicine using nanoformulation-based platforms. The process of tissue regeneration or wound healing is an extremely complex phenomenon. Some of the key factors that affect the tissue regeneration involve (a) hemostasis, (b) inflammation, (c) proliferation, and (d) remodeling. Most biomaterials possess all or some of these characteristics, which are useful for the promotion of tissue regeneration or wound healing. Specifically, some of the polysaccharide biomaterials possess inherent antimicrobial properties, making them an interesting bionano material for tissue regeneration in patients with infectious wounds. Being rich in electronegative and positive functionalities, polysaccharides can be easily complexed with other biomaterials, like synthetic polymers or proteins, to form hybrid nanoscaffolds that can be used for personalized wound healing purposes. Proteins, as they play a part in the central dogma of molecular biology, are an intriguing biomaterial for regenerative medicine. On a molecular level, proteins (such as SF, gelatin, albumin and keratin) are relatively well characterized in terms of their mechanism of action for regenerative medicine or healing application. Moreover, in the last few decades, there has been an exponential increase in the number of reports that suggest proteins as a next-generation biomaterial for both nano drug delivery and regenerative medicine. We observed that many of them have considerable evidence of supporting cell growth corneal healing properties at a molecular level. Hence, their use in ophthalmic applications can be considered to be an interesting avenue. In the present work, we have tried to understand the rationale behind the development of hybrid composite states from various natural biomaterials for personalized tissue regeneration or wound healing.

## Figures and Tables

**Table 1:** Cells directly involved in wound healing process.

SN.	Cells	Proteins Secreted for Cellular Regeneration
1	Platelets	PDGF, TGFβ, EGF
2	Neutrophils	IL-1, IL-6, TNFα, VEGF, PDGFR, TGFβR
3	Fibroblast and myofibroblast	KGF, TNFβ, FGFs, IGF, VEGF, EGF, HGF, PDGFR, TGFβR, FGFRs, IL-1R, TNFαR, SDF-1R
4	Keratinocytes	EGF, IL-1, TGFβ, TNFα, MCP-1, SDF1, HGF, EGFR, R-1R, TGFβR, KGFR, SDF1R
5	Endothelial Cells	FGF, VEGF, PDGF, FGF-2, FGFR, VEGFR, HGFR
6	Macrophages	IL-1, IL-6, TNFα, VEGF, PDGF, EGF, PDGFR, TGFβR, VEGFR

**PDGF:** Platelet-derived growth factor; **TGFβ:** Transforming growth factor beta; **EGF:** Epidermal growth factor; **IL (1 & 6):** Interleukin-1 & 6; **TNFα:** Tumor necrosis factor alpha; **VEGF:** Vascular endothelial growth factor; **PDGFR:** Platelet-derived growth factor receptor; **TGFβR:** Transforming growth factor beta receptor; **KGF:** Keratinocyte growth factor; **IGF:** Insulin growth factor; **HGF:** Hepatocyte growth factor; **FGFRs:** Fibroblast growth factor receptors; **TNFαR:** Tumor necrosis factor alpha receptor; **MCP-1:** Monocyte Chemoattractant Protein-1; **R-1R:** TNF-alpha receptor-like protein; **KGFR:** Keratinocyte growth factor receptors; **VEGFR:** Vascular endothelial growth factor receptor.

**Table 2:** Evolution of CS based materials in bionano science.

SN.	Significant Development	Year	Reference
1	Concept of using charged polysaccharides like CS for drug delivery applications	1989	[12]
2	Development of CS-ethylene oxide-propylene block polymer for protein and vaccine delivery	1997	[13]
3	Oral gene delivery by CS-DNA nanoparticles	1999	[14]
4	Enhancement of insulin by nasal absorption of CS NPs	1999	[15]
5	CS nanocarriers for anticancer drugs	2000	[16]
6	CS nanocarriers for nasal delivery of vaccine	2001	[17]

**Table 3:** Chitosan-nanocomposites and their respective antibacterial activity.

SN.	Nanocomposite	Antibacterial Type	Reference
1	CS coated Ag loaded SiO_2_	*E. coli* and *S. aureus*	[40]
2	CS-Ag NPs loaded nanofiber mats	*E. coli*	[41]
3	CS layered silicate composites	*S. aureus* and *B. subtilis*	[42]
4	Clay chitosan nanocomposite	*E. coli* and *S. aureus*	[43]

**Table 4:** Scaffolds of some of the natural biomaterials and their probable mode of corneal repair in ocular therapy.

SN.	Polymer Type	In Composite State with	Significance	Reference
1	Chitosan	Native hydrogel and membrane	Incorporation of graphene may provide antibacterial properties to nanofibrous scaffold of CS and help in wound healing process.	[66,67]
2	Alginate	Native hydrogel	Improved viability for corneal epithelial cells.	[68,69]
3	Cellulose	PVA hydrogel	Highly transparent, elastic, lubricated and biocompatible material with human corneal epithelial cells (HCE-2)	[70]
	Bacterial Cellulose	PVA composite	Improved mechanical and chemical properties proposed for ophthalmic application	[71]
4	Heparin	Native biomaterial	Established to have corneal wound healing potential. However, nanoscaffold evaluations remain unknown.	[72]
5	Gelatin	Native nanofibers	Improved mechanical properties. The AG-gel consisting of gelatin nanofibers provided improved transparency to formulation.	[73]
		PLLA Nanofibers	Improved compatibility for epithelial cells and keratinocytes and regeneration of corneal stroma.	[74,75]
		PHBV Nanofibers	Helps in epithelial cell proliferation and corneal formation.	[76]
6	Silk fibroin	Native film	Compatible with human limbal stem cell and were helpful in promotion of epithelium formation.	[77]
